# Revealing plant growth-promoting mechanisms of *Bacillus* strains in elevating rice growth and its interaction with salt stress

**DOI:** 10.3389/fpls.2022.994902

**Published:** 2022-09-02

**Authors:** Qurban Ali, Muhammad Ayaz, Guangyuan Mu, Amjad Hussain, Qiu Yuanyuan, Chenjie Yu, Yujiao Xu, Hakim Manghwar, Qin Gu, Huijun Wu, Xuewen Gao

**Affiliations:** ^1^Key Laboratory of Integrated Management of Crop Diseases and Pests, Department of Plant Pathology, College of Plant Protection, The Sanya Institute of Nanjing Agricultural University, Nanjing Agricultural University, Nanjing, China; ^2^Shenzhen Batian Ecotypic Engineering Co., Ltd., Shenzhen, China; ^3^National Key Laboratory of Crop Genetic Improvement, Huazhong Agricultural University, Wuhan, China; ^4^Lushan Botanical Garden, Chinese Academy of Sciences, Jiujiang, China

**Keywords:** rice, cell physiology, cellular interactions, antioxidant enzymes, PGPR, biofilm

## Abstract

Soil salinity is a major environmental stress that has been negatively affecting the growth and productivity of rice. However, various salt-resistant plant growth-promoting rhizobacteria (PGPR) have been known to promote plant growth and alleviate the damaging effects of salt stress *via* mitigating physio-biochemical and molecular characteristics. This study was conducted to examine the salt stress potential of *Bacillus* strains identified from harsh environments of the Qinghai-Tibetan plateau region of China. The *Bacillus* strains NMTD17, GBSW22, and FZB42 were screened for their response under different salt stress conditions (1, 4, 7, 9, 11, 13, and 16%). The screening analysis revealed strains NMTD17, GBSW22, and FZB42 to be high-salt tolerant, moderate-salt tolerant, and salt-sensitive, respectively. The NMTD17 strain produced a strong biofilm, followed by GBSW22 and FZB42. The expression of salt stress-related genes in selected strains was also analyzed through qPCR in various salt concentrations. Further, the *Bacillus* strains were used in pot experiments to study their growth-promoting ability and antioxidant activities at various concentrations (0, 100, 150, and 200 mmol). The analysis of growth-promoting traits in rice exhibited that NMTD17 had a highly significant effect and GSBW22 had a moderately significant effect in comparison with FZB42. The highly resistant strain NMTD17 that stably promoted rice plant growth was further examined for its function in the composition of rhizobacterial communities. The inoculation of NMTD17 increased the relative abundance and richness of rhizobacterial species. These outcomes propose that NMTD17 possesses the potential of PGPR traits, antioxidants enzyme activities, and reshaping the rhizobacterial community that together mitigate the harmful effects of salinity in rice plants.

## Introduction

Rice (*Oryza sativa* L.) is a vital staple food and feed crop that provides nutrition to billions of people around the world. It is usually cultivated as a staple crop in tropical regions (Abbas et al., 2019). Soil salinity is one of the major limiting factors of abiotic stress that decreases rice production. Due to the rising level of soil salinization, crop productivity is limited in many regions of the world (Kumar et al., 2020a). Because of salinity stress, plants can undergo many metabolic, molecular, and physiological changes (Wang et al., 2019; Kumar et al., 2020b, 2021). Salt stress triggers ionic toxicity, osmotic stress, ion imbalance, and decreased water potential, which severely affects plant physiology and metabolism. It mainly affects physiological, morphological, and biochemical processes of plant development and growth (Bistgani et al., 2019). The level of global soil salinization is increasing year by year, and it is estimated that 50% of agricultural land will be affected by salinization by 2050 (Ansari et al., 2019).

Salinity stress affects various aspects of plant development that eventually results in an overall reduction in crop production. For example, it affects seed germination, plant growth and development, spike development, and reproductive growth (Ke et al., 2020; Korenblum et al., 2020). Plant growth and metabolism are severely affected by salt stress because of ion (Na^+^ and K^+^) accumulation. High salt concentrations in soil accumulate simultaneously as Cl^−^ and Na^+^; nonetheless, their impact differs (Abbas et al., 2019; Mengistu, 2020). Increased Na^+^ levels alter soil qualities, such as aeration, water conductivity, and porosity (Compant et al., 2019; Mukherjee et al., 2020). In addition, the cell walls activate osmotic stress and cause cell death in plants with the increase of sodium ions. The chlorophyll content in the leaf is reduced by drought and salinity stresses, which results in reducing the rate of photosynthesis. Stomatal conductance, leaf area, and photosynthetic efficacy may possibly be disturbed by high salinity (Stassen et al., 2020; Hassan et al., 2021; Solangi et al., 2021).

For the sustainable development of agriculture, enhancing the cadmium and salt stress tolerance of plants is therefore of high practical importance (Ali et al., 2022). To overcome this problem, chemical fertilizers and pesticides are widely used in modern agriculture, gradually altering the supply of nutrients, reducing microbial activity, diversity, and deteriorating soil health (Etesami and Glick, 2020; Ali et al., 2021a,b). However, in the natural environment, many microorganisms, including plant growth-promoting bacteria (PGPB) inhabit plants. Plants are exposed to billions of microbes in nature, which form colonies and occupy various compartments of plants, such as endosphere, rhizoplane, rhizosphere, and phyllosphere, which are thus regarded as the secondary genome of plants (Dubey et al., 2019; Kumar et al., 2019). In general, the interaction between plants and microorganisms in the rhizosphere/root zone is essential for plant nutrient acquisition, resistance to various stresses, and plant development (Hubbard et al., 2019; Kong et al., 2021). For in-plant resistance to abiotic stresses, plant relationship with beneficial microorganisms plays an important role (Liu et al., 2020; Sun et al., 2021; Ali et al., 2022). Several studies have evaluated the potential of microorganisms to enhance the growth of host plants under salt stress. A number of studies have reported that beneficial plant bacteria have a complex regulatory mechanism that promote growth and improve host plant damage to salt stress. Plant growth-promoting rhizobacteria (PGPR) increase plant development directly or indirectly by producing growth-promoting traits, for example, 1-aminocyclopropane-1-carboxylate (ACC) deaminase production, and nitrogen fixation (Sarkar et al., 2018; Kumar et al., 2020a).

In addition, rhizosphere bacteria increase plant resistance to salt stress by controlling the effectiveness of photosynthesis, ion homeostasis, osmotic regulation, secondary metabolite accumulation, and plant hormone gene expression signaling pathways (Hassan et al., 2021; Manghwar et al., 2022). Furthermore, various plants possess the natural capability to change soil salinity through metabolism, gene expression, and signal pathway regulation (Daliakopoulos et al., 2016). Salt toxicity is mediated by the formation of antioxidants and the suppression of the generation of reactive oxygen species (ROS) (Abbas et al., 2019). In the natural environment, several crops are recurrently affected by salt stress because it interrupts ecological relations between plants and microorganisms in the soil and inhibits the proliferation of microorganisms in the surrounding environment (Barnawal et al., 2014; Trivedi et al., 2020).

Research has revealed that the bacteria isolated from saline soil, such as *Enterobacter*, *Pseudomonas*, *Arthrobacter*, *Bacillus*, *Chryseobacterium*, *Achromobacter*, and *Ochrobactrum* can enhance plant growth under saline conditions (Sarkar et al., 2018; Kumar et al., 2021). PGPR can help crops survive adverse environments by increasing their development and growth (Kumar and Verma, 2019; Zubair et al., 2019). PGPR are a unique set of microbes that settle/colonize the surrounding plant rhizosphere and promote plant growth in stressful conditions (Kumar and Verma, 2018; Singh et al., 2020). Plant–microbe interactions play a vital role in maintaining soil properties, microbial diversity, and crop productivity under abiotic stresses, including high salinity (Zubair et al., 2019; Kumar et al., 2020b). Several PGPR have been reported worldwide to improve plant growth and development in salt and natural environments (Mukherjee et al., 2019).

The current study aimed to conduct genetic screening and expression analysis to better understand the genetic potential and physiological characteristics of *Bacillus* spp., which enable them to tolerate salt stress. The *Bacillus* spp. were used in the present study to evaluate their potential in alleviating the unfavorable effects of salt stress in rice plants, which showed resistance by regulating their metabolic process under salt stress conditions. The *Bacillus* strains NMTD17 and GBSW22 were found to have a significant PGP ability that enhanced growth and alleviated salt stress on rice plants by regulating salt stress response and plant growth hormones under stress environment. Furthermore, the analysis of relative clusters and structural composition of rhizosphere bacterial clusters through 16S rRNA gene sequencing revealed that the NMTD17 inoculation increased the relative abundance and richness of rhizobacterial species.

## Materials and methods

### Screening of bacterial strains under salt stress

The *Bacillus* strains used in this study were isolated in our laboratory from rhizosphere soil of different plants collected from the Qinghai–Tibetan Plateau, China (Wu et al., 2019). Four strains, *Bacillus* spp. NMTD17, *Bacillus safensis* GBSW22, *Bacillus pumilus* NMSW10, and *Bacillus velezensis* GBSW11 were tested for their growth promotion and salt resistance potential along with model biocontrol strain *B. velezensis* FZB42 as a control on Luria Bertani (LB) agar plates containing various salt (NaCl) concentrations, i.e., 1, 4, 7, 9, 11, 13, and 16%, maintained at 37°C for 4 days (d). The selected strains were then grown in liquid culture to determine their growth pattern *via* the spectrophotometer in terms of optical density (OD_600_) at different time intervals at 37°C for 4 days. The growth curves at various time intervals were used to determine the growth pattern of each strain. The experiment trial was repeated three times.

### Biofilm formation assay under salt stress

The formation of biofilm by microorganisms is an essential feature from which it is possible to analyze their binding to the surface of roots for different functions in their community (Chaves et al., 2020). The selected strains were grown on LB liquid culture in order to know the effect of various salt concentrations on biofilm formation. To achieve an OD_600_ = 1.0, 4 μl, the selected salt-tolerant strains were grown in 20 ml flasks at 37°C. Each bacterial strain was cultured in LB with various salt concentrations, and the resulting mixture was poured onto cluster plates of costar^®^ sterile 12-well cell culture. The cluster plates were tightly closed with parafilm and kept at 37°C for 4 days. A confocal laser scanning microscope (Confocal Microscope Zeiss LSM 780, Japan) was used to evaluate the influence of various salt concentrations on the biofilm formation of each strain. Further, the reactive oxygen species (ROS) were also analyzed under the same conditions (see details in Supplementary material).

### RNA extraction and qPCR

The RNA isolation kit (OMEGA Bio-tek, Inc. Norcross, GA, United States) was used to extract the RNA from different samples as described in (Supplementary material). The sequence of each gene was taken from NCBI, followed by designing primers *via* the primer Quest tool listed in Supplementary Table S1. The *rpsJ* gene was used as a housekeeping gene in *Bacillus* strains, as previously used by Zubair et al. (2019). The cDNA of each sample was used in qPCR to check the gene expression profile. The expression profile of these genes (Supplementary Table S1) in salt-tolerant strains was measured through qPCR [Quant Studio Real-Time Thermocycler (Thermo Fisher Scientific, San Jose, CA, United States)]. The qPCR was programmed with the initial temperature of denaturation 95°C for 30 s, including 40 cycles of 95°C for 5 and 34 s for 60°C. The relative expression levels of the genes were calculated by the method of 2^−ΔΔCT^ as reported by (Ayaz et al., 2021).

### Vigor index and root morphology analysis under salt stress

The effect of salt stress on the germination and growth of rice seedlings was calculated by measuring the vigor index as the formula described by Rasul et al. (2019). The seedlings were then removed from each treatment to measure different root parameters to study the root morphology. The rhizoscanner (EPSON Perfection V700 Photo, Epson America, Long Beach, CA, United States) and WinRHIZO software given by Regent Instruments Co (Sainte-Foy, Quebec, Canada) were used to measure root morphological parameters as described by Rasul et al. (2019). (Detailed description in Supplementary material).

### Salt stress alleviation and plant growth promotion by *Bacillus* strains

The soil was sterilized at 180°C for 30 min and stored in a controlled cold room at 4°C for further use. Seven days old rice seedlings were transplanted into similar-sized pots filled with sterilized soil and kept in a controlled environment in the greenhouse. The PGPR cell cultures [NMTD17, GBSW22, and FZB42 grown overnight to OD_600_ of 1.0 (1 × 107 cfu/ml)] were added into the respective pots. After 1 day, 20 ml salt solutions with various concentrations (0, 100, 150, and 200 mmol) were applied to inoculated PGPR strains and un-inoculated only salt in rice plants. The simple ddH_2_O was used for plants grown as a control (CK). Each treatment was performed in triplicate with three rice seedlings in each pot. After 9 days of inoculation, the effect of salt stress was observed for each treatment. The growth parameters, i.e., fresh/dry weights were measured, and the root morphological parameters were analyzed. The data were used as an indication of growth promotion traits. (See details in Supplementary material).

### Determination of antioxidant enzyme activity

Various stress response parameters were analyzed for bacterial inoculated and control plants in the salt stress environments, for example, catalase (CAT), peroxidase (POD), ascorbate peroxidase (APX), and superoxide dismutase (SOD) 9 days of post-inoculation (dpi) followed by the method of Ayaz et al. (2021). Briefly, fresh leaf samples of 0.3 g were ground in a phosphate buffered solution (PBS) of pH 7.8 and 1 mM EDTA in an ice bath, followed by centrifugation for 30 min and 12,000 rpm at 4°C. Enzyme extracts were made from the supernatant using ddH_2_O as a control. The absorbance activity was recorded at 240 nm for CAT, 470 nm for POD, 560 nm for SOD, and 290 nm for APX, and ddH_2_O was used as a control. The commercial kit (Nanjing Jiancheng Bioengineering Institute, China) was used to determine the APX concentrations.

### RNA extraction and gene expression analysis

To find the relative expression of the above salt stress response parameters, the genes *Ossamdc2*, *Osdreb1f*, *Oserebp2*, *Oslea3-1*, *Oserf104*, and *Oscyp89g1*, and the actin gene *OS03G0836000* were used. For this, the selected gene sequences were taken from NCB1, followed by designing primers through the PrimerQuest tool; primers are listed in Supplementary Table S2. For RNA extraction, the fresh leaves of plants were harvested from PGPR inoculated plants grown in a salt environment for 7 days using the TRizole method. The Applied Biological Materials Inc. (abm^®^, Beijing, China) 5 × All-In-One RT Master Mix (with AccuRT Genomic DNA Removal Kit) kit was used for cDNA synthesis. The qPCR was performed to analyze the expression profile of selected genes in rice plants through Quant Studio Real-Time Thermocycler (Thermo Fisher Scientific, San Jose, CA, United States). The PCR machine was programmed using the following steps: i.e., initial denaturation at 95°C for 30 s, including 40 cycles of 95°C for 5 s, and 34 s at 60°C. Finally, relative quantification was performed according to the comparative C method of 2^−ΔΔCT^ as described by (Liang et al., 2022).

### DNA extraction, 16Sr DNA gene sequencing, and bioinformatics analyses

Rhizosphere soil samples were collected from PGPR inoculated and un-inoculated rice plants grown under various salt treatments (0, 100, 150, and 200 mmol). The genomic DNA was isolated using the applied protocol TIANamp Soil DNA Kit [TIANGEN Biotech (Beijing) Co., Ltd.]. The V5–V7 bacterial portions of the 16S rRNA gene were amplified using primers (799F: AACMGGATTAGATACCCKG, and 1193R: ACGTCATCCCCACCTTCC), as described in Supplementary material. The refined amplicon was combined in equimolar amounts and sequenced with an Illumina MiSeq platform following paired-end pairing (2 × 300) according to Meige Biotechnology Co., Ltd. (Guangzhou, China) standard protocols (Illumina, San Diego, CA, United States). The fastq-formatted sequences were processed and analyzed using QIIME2 (ver. 2022.01). Sequence read quality, denoising, and filtering were examined by following the procedure of Han et al. (2020). Briefly, fastq-formatted sequences with primers, quality scores < 28, and read length < 300 bp were removed. The diversity metrics of within-sample (alpha-diversity; Observed, Shannon, Chao1, ACE, and Simpson index) and between samples (β-diversity; Bray-Curtis matrix) were calculated using the R package vegan (version 2.1). Spearman’s correlation coefficients were used to find correlations among the bacterial community at the genus level with growth promotion and plant antioxidant activity parameters.

### Statistical analyses

All the experiments were carried out in a completely randomized design. The data were expressed in ± standard deviations (SD) of three replicates (*n* = 3). The means were calculated using Tukey’s HSD test at *p* ≤ 0.05 after ANOVA. IBM SPSS Statistics 21.0 was used to conduct all statistical analyses of the data. The graphical illustrations were made with Origin graphical and analysis software (Version 2022, OriginLab Corporation, Northamptom, MA, United States).

## Results

### Bacterial growth screening under various salt concentrations

**Figure 1:**
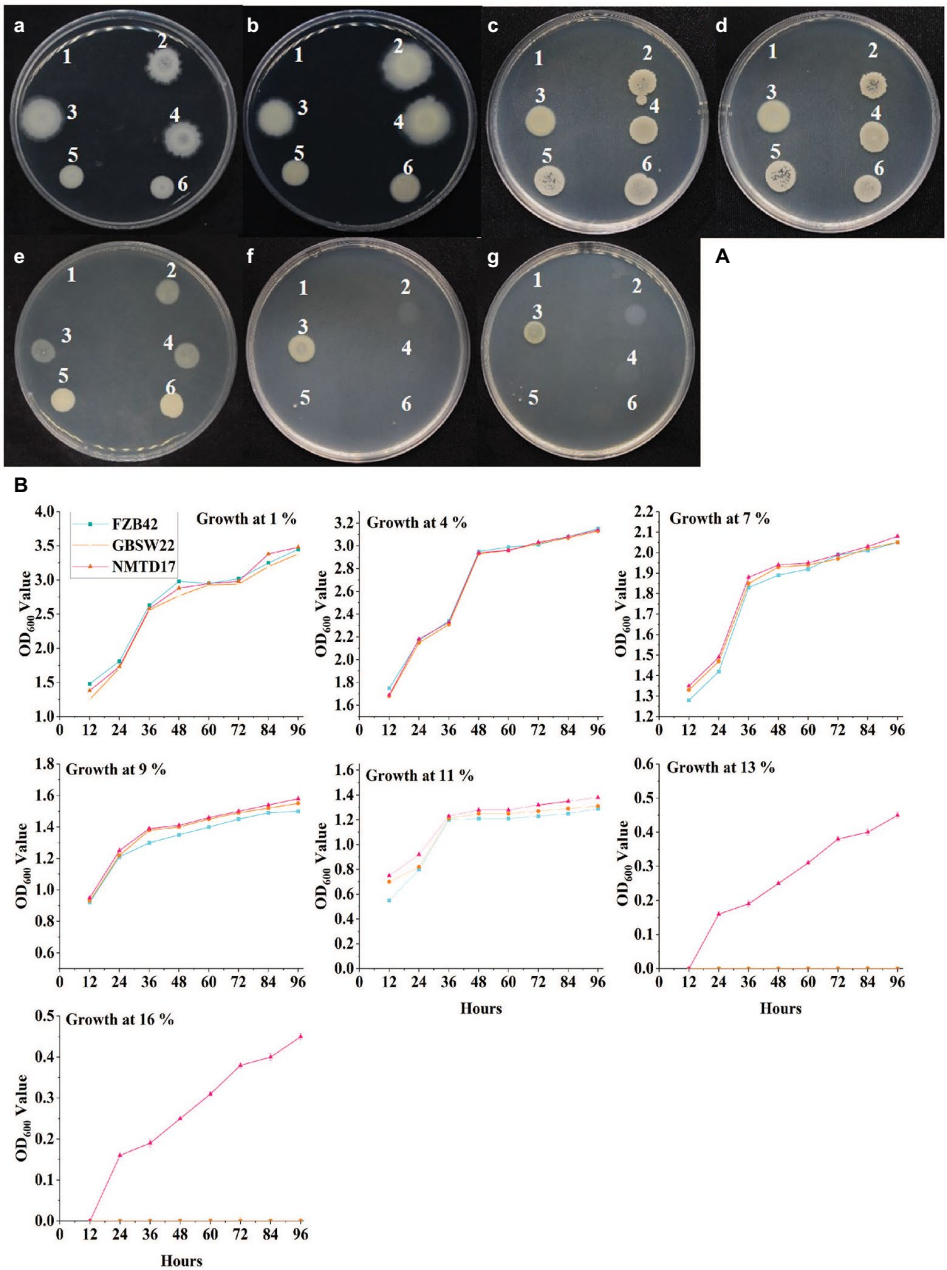
The *Bacillus* spp. grown on LB media with salt stress incubated for 96 h at 30°C. **(A)** The growth of strains on solid LB media with different salt concentrations (a-g represent 1, 4, 7, 9, 11, 13, and 16%, respectively). Each number showing *Bacillus* strain: (1) CK, (2) FZB42, (3) NMTD17, (4) GBSW22, (5) NMSW10 and (6) GBSW11. **(B)** The graphical representation of the optical density of each strain at the same salt concentrations (1 to 16%) at different time intervals measured by spectrophotometer.

The PGPR strains isolated from harsh environments were screened for their growth potential under different salt concentrations (1, 4, 7, 9, 11, 13, and 16%) on LB medium along with a known salt-sensitive *B. venzelisis* FZB42 strain. Most of the strains, including FZB42 and GBSW22 were able to grow on LB medium containing up to 11% salt concentration. The strain NMTD17 was able to grow up to 13 and 16%, showing the highest resistance under salt stress conditions compared to control (Figure 1). All three strains were then grown in LB liquid medium containing the same salt concentrations (1–16%) as mentioned above, and the growth pattern of each strain was evaluated through OD_600_ calculation at 600 nm by using a spectrophotometer. The OD_600_ was calculated at different time intervals, and growth parameters were noted, which showed that the growth curve for strain NMTD17 continuously increased in a linear method up to 96 h (Figure 1). The salt-tolerant strain NMTD17 exhibited the highest growth at 16% salt treatment, followed by GBSW22. In comparison, the growth curve of FZB42 showed a non-significant growth pattern at 16% post-inoculation up to 96 h.

### Biofilm formation assay under salt stress and ROS production

**Figure 2:**
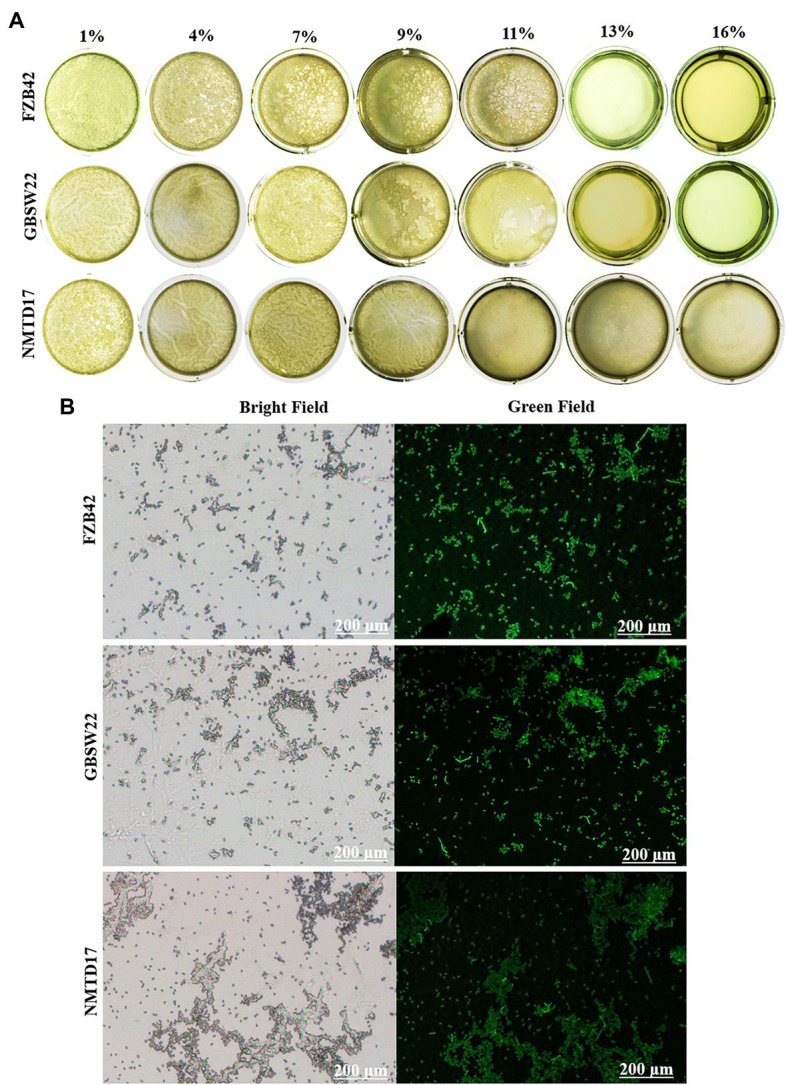
The biofilm formation of *Bacillus* strains grown under different salt conditions (1, 4, 7, 9, 11, 13, and 16%) up to 96 h at 37°C. The biofilm formation ability was observed in selected *Bacillus* strains **(A)**. The higher amount of green fluorescence in the FZB42 strain cultured for 4 days at 37°C indicates a higher quantity of reactive oxygen species (ROS). When compared to GBSW22 and FZB42, the reduced fluorescence indicates that NMTD17 cells produce less ROS **(B)**.

The results of the three strains’ biofilm-forming capabilities under various saline environments (1, 4, 7, 9, 11, 13, and 16%) at 37°C with multiple time intervals up to 96 h exhibited that all three strains were able to form the finest biofilm structure up to 11% salt concentration. However, the potential of FZB42 to make biofilm at 11% was reduced and finished under high saline conditions of 13–16% up to 96 h (Figure 2A). NMTD17 was observed to be the strong strain to form biofilm continuously up to 16% and enclosed the full well surface at 96 h after inoculation, followed by GBSW22. The strains FZB42 and GBSW22 were unable to generate appreciable biofilm structure at 16% salt concentration at 96 h after inoculation. In addition, selected strains were grown in 16% LB media at 37°C for 4 days to determine their ROS levels. The data showed that salt-tolerant strains NMTD17 and GBSW22 sustained lower levels of ROS-stained cells observed under the microscope. Whereas the strain FZB42 exhibited a considerable rise in the amount of ROS production when grown in the same conditions (Figure 2B).

### Relative expression profiling of predicted genes through qPCR

**Figure 3:**
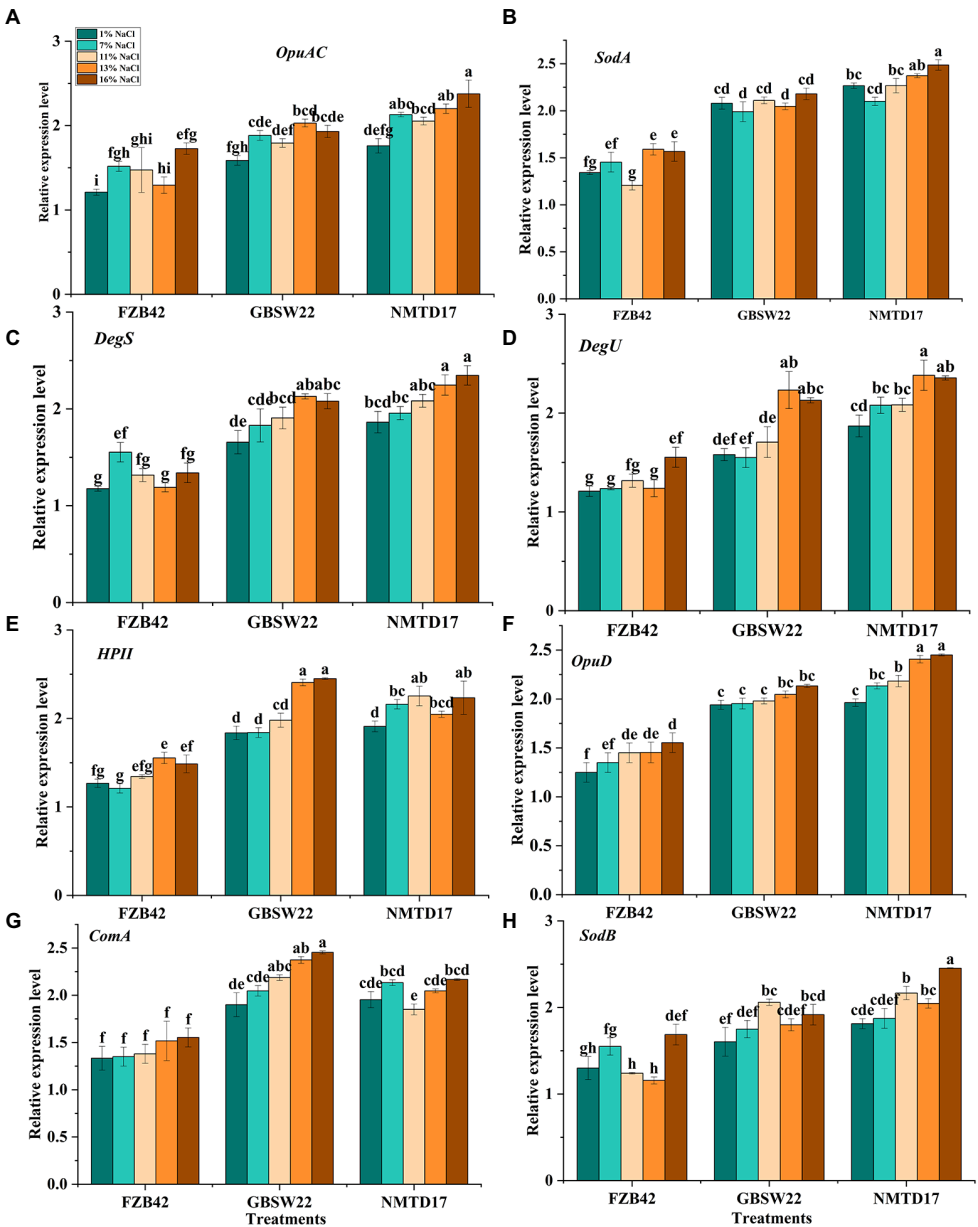
Relative expression levels of various *Bacillus* strains grown under salt and normal conditions for 96 h. **(A)** *OpuAC,* **(B)** *SodB,* **(C)** *DegS,* **(D)** *DegU,* **(E)** *HPII,* **(F)** *OpuD,* **(G)** *ComA*, and **(H)** *SodA*. Vertical bars on graphs indicate the standard deviation of the mean (n = 3). Tuckey’s HSD test was used to recognize a significant difference at *p* ≤ 0.05 between the treatments.

The expression profile of important genes involved in salt resistance was studied in NMTD17, GBSW22 and FZB42 grown in various salt treatments (1, 7, 11, 13, and 16%) for 4 days. The results showed a linear up-regulation in the expression of salt-resistant genes in NMTD17 and GBSW22 as compared with control. The relative expression of salt-resistant genes in FZB42 was observed up to 11% salt treatment. However, by increasing saline conditions, i.e., 13 and 16%, FZB42 was unable to exhibit the expression of salt-resistant genes (Figure 3). The high expression of selected salt-resistant genes was noticed in NMTD17 followed by GBSW22 in linear order by increasing salt treatment from “11% to 13 and 16%.” The genes (*DegU* and *DegS*) involved in the signal transduction pathway mitigating stress response also revealed elevated expression levels for NMTD17 and GBSW22 strains. The responsible superoxide dismutase genes (*SodA* and *SodB*) were noticed to be highly expressed in respective treatments under saline conditions. The glycine betaine genes (*OpuAC* and *OpuD*) responsible for osmotic stress were also increased in NMTD17, followed by GBSW22 under salt conditions. The *HPII* gene playing an important role in catalase regulation under saline conditions, was observed to be highly expressed in NMTD17. The high expression of the *ComA* gene involved in quorum-sensing regulation, was also observed in NMTD17 and GBSW22. The overall results exhibited that all the selected salt-resistant genes showed a significant relative expression level in high salt-resistant strains, as shown in Figure 3.

### Seedling growth and root morphological parameters

The inoculation of selected strains promoted seedling development and root morphological factors under different saline conditions (0, 100, 150, and 200 mmol). The seedling growth in vigor index (VI), which is a measure of total germination (%) and total length of seedlings, which was found to be highest in rice seedlings treated with NMTD17 (VI = 1,210) under high saline conditions (200 mmol) that was significantly greater than control, as well as GBSW22 and FZB42 treatments as shown in Supplementary Figure S1. Seedlings inoculated with GBSW22 showed moderate VI (VI = 1,157) growth under 200 mmol saline conditions. At normal condition (0 mmol), the rice seedlings inoculated with all selected *Bacillus* strains NMTD17, GBSW22, and FZB42 demonstrated the significant VI (VI = 1,211, 1,157, and 1,153 respectively) as compared to control (Supplementary Figure S1).

Furthermore, root morphological analysis revealed that the root morphological parameters were significantly improved under control and salt stress treatments when inoculated with selected strains. However, NMTD17 exhibited the highest impact on seedling root morphology, such as root volume, area, length, diameter, and number of root tips under high salt stress (200 mmol) compared to control seedlings grown under the same condition. When compared to the control under normal conditions (0 mmol), seedlings treated with all three bacterial strains significantly improved root morphological factors (Supplementary Figure S2).

Furthermore, the seedling and plant root morphology, including total root length, root tips, volume, diameter, and surface area were analyzed. PGPR strain NMTD17 increased plant root morphological parameters by 2–3-fold as compared to controls plants. Root diameter, surface area, and tips were increased significantly in the case of NMTD17-inoculated plants, followed by the plants inoculated with GBSW22 and FZB42 (Supplementary Figure S3). Thus, the strain NMTD17 was found to be more effective at reducing the risk of high salt stress treatment (200 mmol) compared to GBSW22 and FZB42.

### The growth of rice plants under salt stress

The plant growth promotion characteristics showed significant improvements under both normal and salt stress conditions when inoculated with PGPR strains. As mentioned in above findings, the strain NMTD17 exerted the highest impact on plant growth parameters, i.e., plant shoot length and fresh and dry weight were significantly increased by NMTD17 under salt stress compared to control plants (Supplementary Figure S4). Plants inoculated with GBSW22 showed substantial growth improvement as well, whereas FZB42-inoculated plants were unable to tolerate high salt stress (200 mmol). In normal conditions, i.e., 0 mmol, the selected strains significantly enhanced shoot length and fresh and dry weight of rice plants in comparison to the control. Whereas no significant difference was found among the selected strains for different growth-promoting factors. Moreover, the increase in salt stress condition, i.e., 100−200 mmol negatively affected the growth of un-inoculated rice plants. The NMTD17 presented the most beneficial effect on rice seedlings by alleviating the negative effect of increased saline condition. The overall data showed that the loss caused by salt toxicity in rice plants was observed to be alleviated in the presence of selected strains NMTD17 and GBSW22 (Supplementary Figure S4).

### Antioxidant enzymes activity under salt stress in rice plants

The increased soil salinity effectively modulates the microbial community, plant enzyme activity, and soil health. Thus, the antioxidant activity of different enzymes (POD, CAT, SOD, and APX) was examined in rice plants grown under different salt concentrations. The bacterial-inoculated rice plants had higher POD activity than the control plants under salt treatment. The POD activity increased by 20.22, 45.35, and 50.45% with FZB42, GBSW22, and NMTD17, respectively, in salt-stressed plants. It increased 10.45, 15.39, and 20.85% with FZB42, GBSW22, and NMTD17, respectively, in the control plants, as shown in Supplementary Figure S5a. The inoculated plants exhibited higher SOD activity as compared to control plants. Compared to control, the SOD activity was increased by 25.75, 45.46, and 52.68% in inoculated plants under salt stress, followed by 12.25, 20.65, and 35.34% with FZB42, GBSW22, and NMTD17, respectively, in control plants without salt stress (Supplementary Figure S5c). The activity of CAT was increased due to salt stress in rice plants, and it was greater under the salt condition as compared to the control. The plants inoculated with FZB42, GBSW22, and NMTD17 showed higher CAT activity 25.36, 35.75, and 55.57%, respectively, as compared to control plants followed by 15.65, 18.36, 20.45%, respectively, compared to control plants as shown in Supplementary Figure S5b. Similarly, the activity of APX increased due to salt stress in rice plants, where the inoculated plants showed higher APX activity as compared to control plants under salt stress. The APX activity was increased by 45.25, 50.63, and 58.35% in inoculated plants and 13.36, 15.45, and 19.68% in control plants for FZB42, GBSW22, and NMTD17, respectively (Supplementary Figure S5d). In general, the results revealed that as the salt concertation was increased in rice plants, the antioxidant activity was significantly enhanced with bacterial inoculation as compared to control conditions (Supplementary Figure S5).

### Relative gene expression analysis

**Figure 4:**
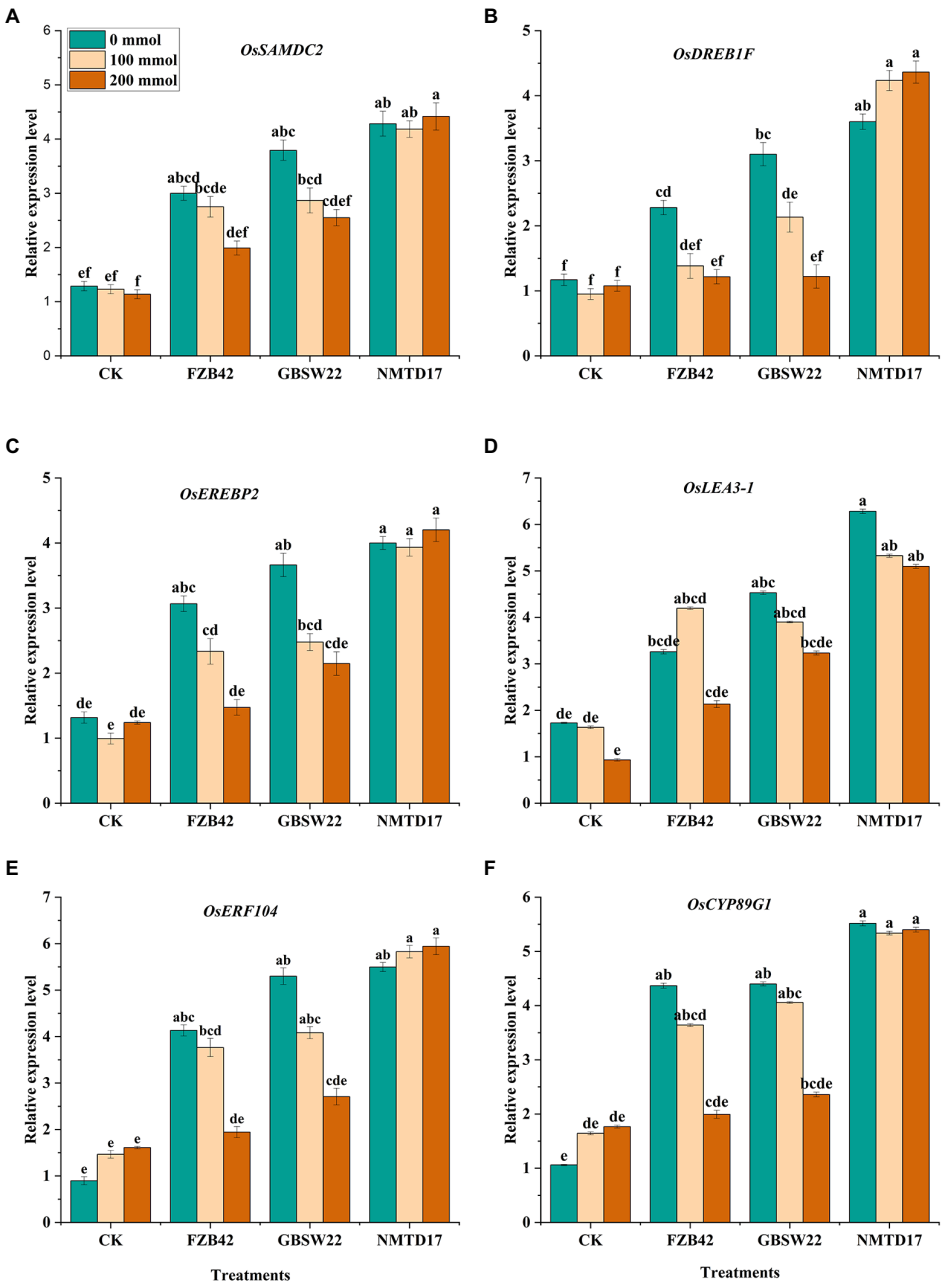
Relative expression levels of six possible DEGs in inoculated and un-inoculated rice plants under salt stress and control conditions **(A)** *Ossamdc2,* **(B)** *Osdreb1f,* **(C)** *Oserebp2,* **(D)** *Oslea3-1* **(E)** *Oserf104,* **(F)** *Oscyp89g1*. The rice plants were subjected to different treatments. Vertical bars on graphs indicate the standard deviation of the mean (*n* = 3). Tuckey’s HSD test was used to recognize a significant difference at *p* ≤ 0.05 between the treatments.

The relative expression profiling of various differentially expressed genes (DEGs) related to salt stress, including *Ossamdc2*, *Osdreb1f*, *Oserebp2*, *Oslea3-1*, *Oserf104*, and *Oscyp89g1* was observed to be highly stimulated in inoculated rice plants under salt stress. We observed that DEG expression levels under various salt circumstances were not the same as that of the control rice plants. After salt treatment, the plants treated with the highly halophilic strain NMTD17 showed the highest up-regulation of all the six salt stress-responsive genes, followed by GBSW22 and FZB42 compared to control plants (Figure 4). The 4–5-fold up-regulation of these genes was found in rice plants inoculated with NMTD17 grown under high salt stress conditions as compared to control plants. In addition, GBSW22 exhibited the higher expression of these genes, followed by FZB42. It was observed that the rice plants inoculated with halophilic strains NMTD17 and GBSW22 showed higher expression of salt stress-related DEGs under saline conditions, which might play important roles in rice tolerance to high salinity.

### Microbial community associated with rice rhizosphere

**Figure 5:**
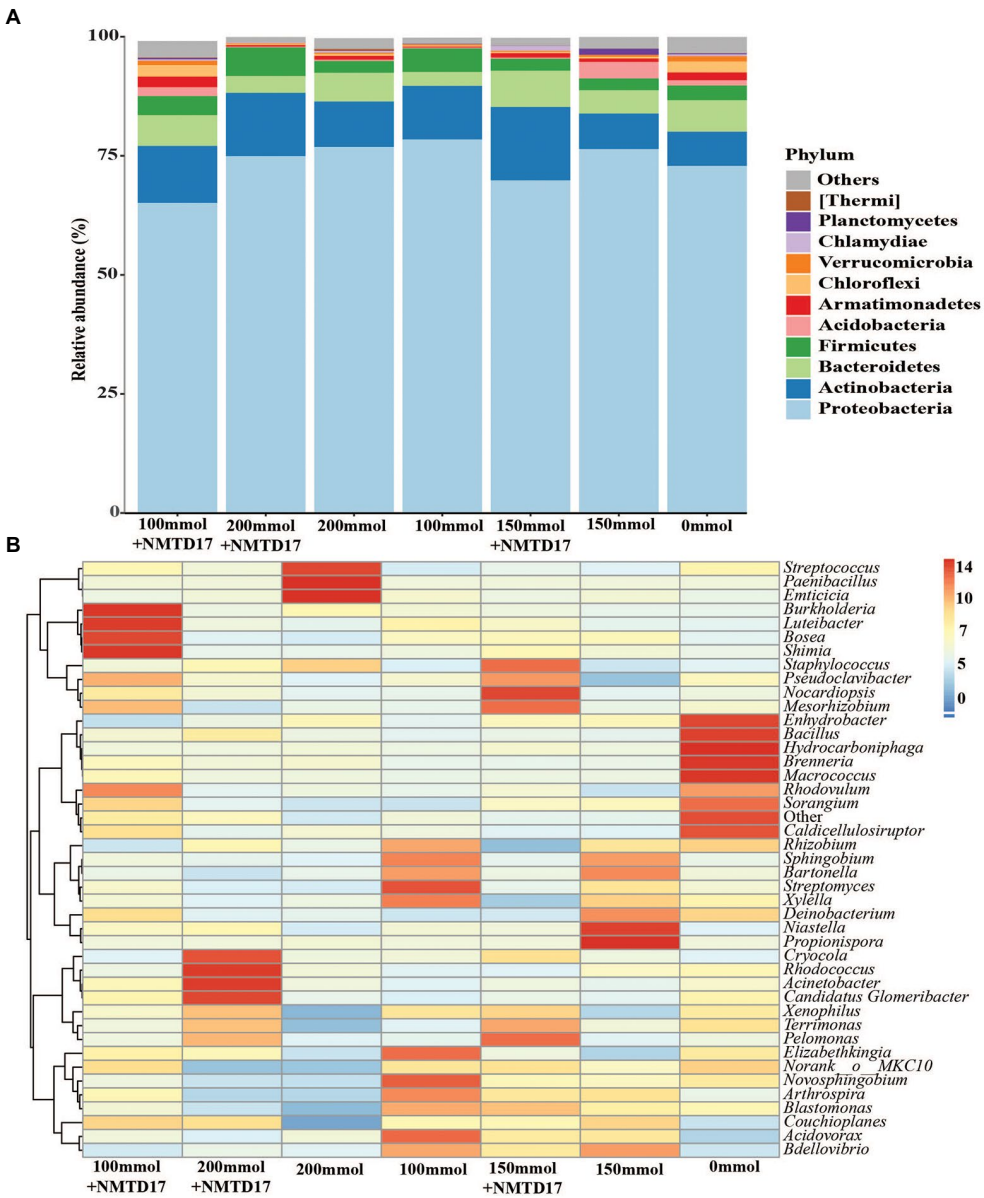
Taxonomic composition of the microbial communities in different inoculated and un-inoculated rice rhizosphere soils. **(A)** Microbial compositions at the phylum level. **(B)** Heatmap of compositions showing 43 microbial communities at the genus level.

The relative abundance of the dominant bacterial community in inoculated and non-inoculated rice rhizosphere was determined using Illumina sequencing and the 16S ribosomal-RNA (rRNA) sequencing method. At the phylum level, the classification of the high-quality fragments revealed changes in bacterial communities among the selected treatments (Figure 5). A total of 28 bacterial phyla were found across all samples: the twelve with a relative abundance > 1.0%, as shown in Figure 5A. *Proteobacteria* was the most prevalent phylum in all treatments, with a relative abundance of 78.26%, *Actinobacteriota* 25.94%, *Bacteroidetes* 7.65%, *Firmicutes* 30.22%, and *Armatimonadetes* 2.33%. Both the inoculated and un-inoculated rice rhizosphere soils showed that *Proteobacteria* was the dominant phylum among all bacterial communities. In addition, the heatmap was constructed, which revealed significant differences in relative abundances of taxa among rice rhizosphere soil samples (Figure 5B). The analysis at the genus level exhibited that the genera *Enhydrobacter*, *Bacillus*, *Hydrocarboniphaga*, *Brenneria*, *Macrococcus*, *Sorangium*, and *Caldicellulosiruptor* were highly abundant in control. Whereas the genera *Burkholderia*, *Luteibacter*, *Bosea*, *Shimia*, *Pseudoclavibacter*, *Nocardiopsis*, *Mesorhizobium*, *Cryocola*, *Rhodococcus*, and *Actintobacter* had a high abundance in inoculated followed by *Streptococcus*, *Paenibacillus*, *Emiticicia*, *Niastella*, and *Propionispora* in un-inoculated soil. The data revealed that a high abundance of genera was found in inoculated and un-inoculated rice rhizosphere soil under salt stress as compared to control.

### Diversity matrix of bacterial community

**Figure 6:**
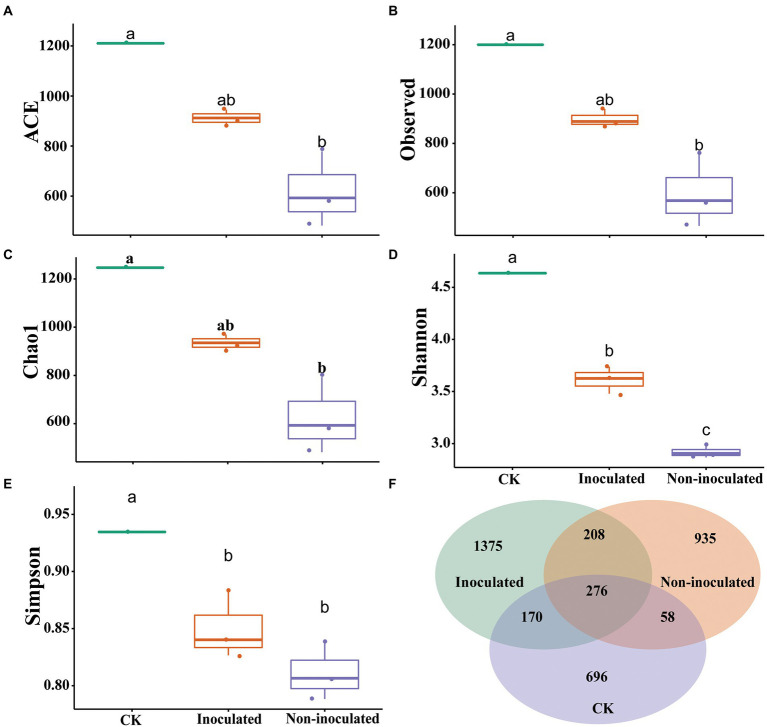
Analysis of the bacterial communities in various inoculated and un-inoculated rice rhizosphere soils. The box diagram of alpha- and beta-diversity of the inoculated and un-inoculated rice rhizosphere soil in groups **(A)** ACE, **(B)** Observed value, **(C)** Chao1 index, **(D)** Shannon index, **(E)** Simpson index, **(F)** Venn diagram of bacterial communities of different inoculated and inoculated rice rhizosphere soils in groups based on OTUs.

The evaluation of the α-diversity of rhizosphere bacterial community among each treatment showed significant differences in microbial diversity. The alpha-diversity and beta-diversity, such as Observed index, Shannon index, Simpson index, Chao1 index, and ACE diversity of the microbial community in rhizosphere soil isolated from inoculated and un-inoculated treatments were observed. The outcomes revealed a significant difference in the microbial community in inoculated, un-inoculated, and control rhizosphere soils (Figures 6A–E). Significant variances were also observed for the rhizobacterial community from rice rhizosphere soil treated with different salt concentrations. These findings suggested that the rhizospheric bacterial community diversity and relative abundance of the bacterial microbiomes within the rhizosphere might be influenced by salt stress. Besides, in inoculated rhizosphere soil, the bacterial community increased due to *Bacillus* strain NMTD17, which is highly resistant to salt stress. To validate these outcomes, the Venn diagram was used to compare and contrast the bacterial communities of all treatments based on operational taxonomic units (OUTs; Figure 6F). The overall number of common OTUs was 276, accounting for 48.25% of all OTUs detected (i.e., 1,375). The shared OTUs indicated that microbial community existed in all respective treatments.

### Principal coordinate analysis (PCoA) and correlation of bacterial communities with plant growth promotion

**Figure 7:**
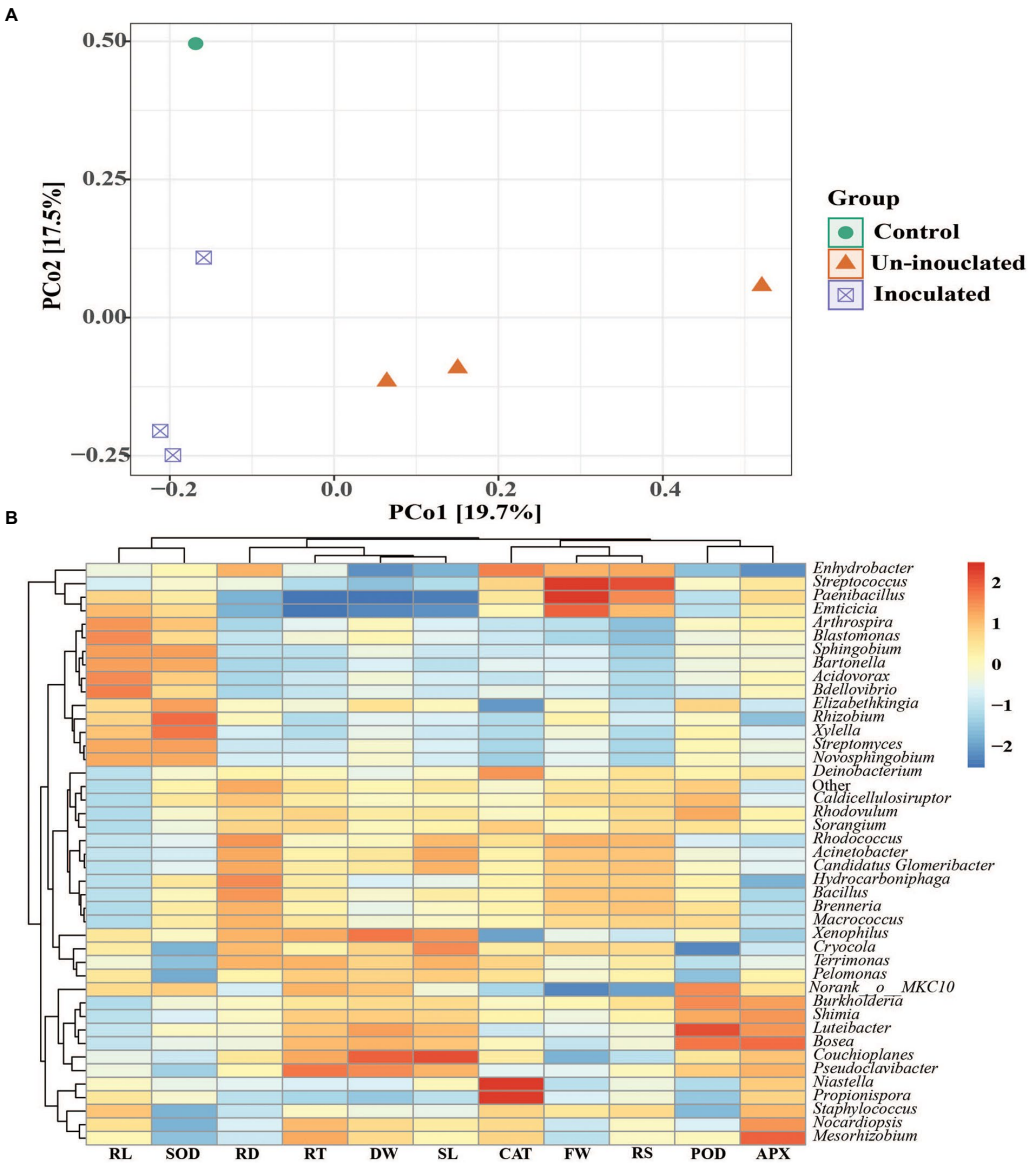
**(A)** The Principal coordinate analysis (PCoA) ordinations of bacterial community composition based on total OTUs using the Bray–Curtis matrix from the rhizosphere of rice under inoculated and un-inoculated plants. **(B)** Spearman’s correlation coefficients between the relative abundance of dominant bacterial community at genera level with plant growth and antioxidant activity parameters. Root length (RL), superoxide dismutase (SOD), Root diameter (RD), Root tips (RT), Dry weight (DW), Shoot length (SL), catalase (CAT), Fresh weight (FW), Root surface (RS), Peroxidase (POD), ascorbate peroxidase (APX).

To understand more details, we conducted the Bray–Curtis distance metrics using principal coordinate analysis (PCoA), which indicated the differences in microbial communities of inoculated and un-inoculated rice rhizosphere soil (Figure 7A). The inoculated and un-inoculated rhizosphere soil in rice showed a similar microbial community structure. However, the inoculated rhizosphere soil was separated according to salt concentration level. The results demonstrated that the samples with an inoculated and un-inoculated rhizosphere soil were separated as compared to control due to salt stress, indicating that microbial communities and structural sequence were different in inoculated and un-inoculated rhizosphere soil.

The relative abundances of dominating bacteria at the genus levels were calculated using Spearman’s correlation coefficients, and growth promotion parameters were identified at the jointing stage. The dominating bacteria, rice growth and antioxidant activity displayed a general positive or negative correlation. In inoculated and un-inoculated rice rhizosphere soil, relative abundances of *Streptococcus*, *Paenibacillus*, *Propionispora*, *Rhizobium Couchioplanes*, and *Niastella* at the genus level were positively correlated (*p* < 0.05) with rice growth promotion and enzymatic activity (Figure 7B).

## Discussion

Salinity stress is one of the important abiotic factors affecting growth and production of crops, including rice, which necessitates appropriate sustainable agricultural management (Akram et al., 2019; Kumar et al., 2021). Many plant growth-promoting rhizosphere bacteria are known to mitigate the hazardous effects of abiotic stresses, including salinity and heavy metals through a variety of direct and indirect processes and eventually enhance crop development and yield (Choudhary, 2012; Hussain et al., 2019). However, the effectiveness of PGPR in reducing plant salt stress in plants has yet to be completely investigated. Therefore, in the present study, the *Bacillus* strains were screened for salt tolerance *in vitro* and the prospective salt-tolerant PGPR strains with desired traits were then employed in soil–plant systems under salt stress conditions. The growth curves at different time intervals up to 96 h of growth indicate the high potential of these strains against salt stress (Figure 1). Among these, the NMTD17 strain exhibited a higher potential to grow at a high salt concentration (16%) as compared to GBSW22 and FZB42. Previously reported PGPR strain FZB42 was also used that could effectively grow up to 11% at salt stress conditions but failed to grow as the concentration was increased from 13 to 16% (Wu et al., 2019).

The role of PGPR in biofilm formation in rhizosphere soil and root surface colonization is being increasingly recognized as a key feature for successful survival mechanisms (Ansari and Ahmad, 2018; Abbas et al., 2019). Biofilm is a collection of bacteria in a self-secreted environment that allows bacterial colonies to exist and produce secondary metabolites in extreme environments (Marsden et al., 2017). After 4 days’ inoculation in LB media, the maximum biofilm formation was found in NMTD17 under salt stress, whereas GBSW22 and FZB42 developed biofilm structures up to 11% saline condition, and it lost biofilm ability as the salt concentration was raised from 13 to 16% (Figure 2A). Research has reported that bacterial biofilm structures are distorted under stress (Feldman et al., 2016), but there is very little evidence of biofilm development under severe salt stress conditions. The halophilic PGPR strains employed in the present study exhibited a unique biofilm-forming ability that allows them to persist and generate the essential metabolites under salt stress. This characteristic is related to the bacterial ability to colonize plant roots and help them cope with salt stress. In addition, ROS are dominant free oxy-radicals that can be generated in response to biotic and abiotic stresses (Ramu et al., 2016; Ayaz et al., 2021). The selected bacterial strains were cultured under salt conditions for 96 h. The salt-sensitive strain FZB42 produced the high ROS, as demonstrated by green fluorescence emitted by the bacterial cells, showing a higher level of cellular disruption (Figure 2). In contrast, the NMTD17 and GBSW22 strains produced the least ROS labeled cells, showing that these strains can withstand salt stress agreeing to (Zubair et al., 2019).

Furthermore, important genes involved in membrane transport, lipid metabolism, fatty acid regulation, and cell signaling, as well as those involved in combating salt stress were analyzed to compare their expression in FZB42, GBSW22, and NMTD17. The following genetic characteristics have a significant influence on abiotic stress tolerance in bacteria that have already been examined (Allen et al., 2009; Zubair et al., 2019). Under salt treatments, the expression level of genes associated with salt stress tolerance in all three *Bacillus* strains revealed an up-regulation of selected genes in PGPR strain NMTD17 and GBSW22, which might be a key cause of growth capacity to survive in high salinity conditions. The expression of all these genes decreased in FZB42 as salt concentration was increased, which might be the reason for its inability to survive in high salt concentrations. The expression level of the quorum-sensing regulator gene *ComA*, which acts as a transcriptional activator for several significant physiological responses in bacteria, also helps salt-tolerant strains survive under salt stress conditions (Dogsa et al., 2014). The signal transduction pathway genes (*DesS* and *DegU*) had higher expression levels in NMTD17 and GBSW22, suggesting that these genes can change the response to abiotic stress or a changing environment. This is consistent with the previous finding that these genes play a significant role in the *Bacillus Pumilus* 7P strain under salt stress (Helmann et al., 2003). When these genes are overexpressed in NMTD17 and GBSW22, the strains are better able to perceive and detect the salt stress signal, but FZB42 only minimally increases the expression of these genes growing under high salt stress conditions. Glycine betaine encoding genes (*OpuD* and *OpuAC*) are key osmo-protectants and are involved in osmotic stress management. In NMTD17, the *OpuAC* gene revealed the greatest expression level (4-fold), followed by GBSW22 (Figure 3). Our results are agreeing by the fact that these transcription features are active in response to abiotic stress (Helmann et al., 2003).

We also analyzed different plant growth attributes, such as root length, dry weight, fresh weight, height, antioxidant enzymes, and microbial quantity. In general, plant growth characteristics reduced as the salt concentration in control plants was increased. However, compared with the control, the plant growth attributes of the inoculated plants were significantly enhanced (Supplementary Figure S4), as supported by (Zhang et al., 2018; Ansari et al., 2019). Salt stress has been linked to an imbalance in plant nutrition and osmosis, resulting in lower growth and photosynthesis (Khan et al., 2019). The application of the *Bacillus* NMTD17 strain proved that it could restore normal physiological characteristics more effectively, followed by GBSW22 and FZB42 as compared to control in rice plants under salt stress. Similarly, Zhang et al. (2018) described the restoration of plant physiological functions through bacterial inoculation.

The use of PGPR to enhance plant development has received much attention because PGPR can help plants grow better under stress conditions (Etesami and Maheshwari, 2018; Li et al., 2020). Inoculating *Pseudomonas* sp. into pea plants under drought stress has been observed to promote the development of pea plants (Sarkar et al., 2018). Similarly, in the present study, the PGPR *Bacillus* strains NMTD17 and GBSW22 improved rice plant development under salt stress *via* increasing seed vigor index (Supplementary Figures S1, S2). Under normal conditions, the selected PGPR strains showed the highest results for all seedling growth promotion factors, but this is the first report of the FZB42 strain having no significant effect on plant development when exposed to high salt (200 mmol). However, it is well proven that *Pseudomonas* and *Bacillus* strains have a favorable influence on seedling and young plant vigor index and root morphological characteristics (Rasul et al., 2019; Kumar et al., 2021). However, we found a significant difference in the root morphological and vigor index factors of rice seedlings and rice plants under salt stress after inoculation with highly halophilic *Bacillus* strains NMTD17 and GBSW22. The fresh and dry weight of root and shoot length of inoculated plants increased significantly under salt stress when compared to control plants (Supplementary Figure S3), which is in accordance with (Ansari and Ahmad, 2018; Abbas et al., 2019).

The outcomes of this investigation clearly reveal that plants treated with *Bacillus* strains have significantly increased the activity of antioxidant enzymes (Supplementary Figure S5). Under salinity stress, the studies have discovered that bacterially inoculated plants have higher antioxidant enzyme activity (CAT, SOD, POD, and APX) than control plants (Narayanasamy et al., 2020; Kumar et al., 2021). The findings of this study demonstrate that as the concentration of NaCl in rice plants increased, antioxidative enzyme activities like CAT and SOD were also increased (Numan et al., 2018; Mubeen et al., 2022). Increased NaCl content was closely associated with higher antioxidant enzyme activity in rice (Khan et al., 2017; Numan et al., 2018). Plants have evolved an enzymatic defense mechanism to alleviate the effects of salt stress. The findings imply that at greater NaCl doses, increased antioxidant activity is necessary to protect plants from oxidative stress caused by salt stress (Li et al., 2020).

Furthermore, stress-related genes were also studied to explain the improved resistance to salt stress. Using qRT-PCR, we confirmed the expression levels of six genes, including *Ossamdc2*, *Osdreb1f*, *Oserebp2*, *Oslea3-1*, *Oserf104*, and *Oscyp89g1* (Figure 4). The NMTD17 and GSW22 strains upregulated the expression levels of *Oscyp89g1* and *Oserf104* genes, which are members of the *CPY* and *ERF* families and may be crucial in stress-related responses (Yao et al., 2018; Yu et al., 2018; Zhang et al., 2020). Through binding to the DRE/CRT element (G/ACCGAC), the transcription factor gene *Osdreb1f* has been found to increase rice salt tolerance by promoting the expression of many stress-related genes (Wang et al., 2008). A transcription factor that regulates a receptor-like kinase gene *Oserebp2*, which is a negative regulator of the NaCl stress response, was found to be involved in rice NaCl tolerance (Serra et al., 2013). S-adenosylmethionine decarboxylase and stress-induced protein kinase genes were encoded by *Ossamdc2*. The ROS scavenging pathway of these genes was postulated to be implicated in rice salt tolerance (Ouyang et al., 2010; Shah et al., 2021). The relative expression level further supports our findings that *Bacillus* strains have the ability to regulate the genes mentioned above under salt stress. We confirm that the *Bacillus* strains NMTD17 and GSW22 can alleviate salt toxicity in rice by owning genes that also improve growth and regulate the expression level of stress-responsive genes and improve plant development under salt stress.

Numerous studies have shown that the PGPR application can strongly impact the bacterial community (Song et al., 2021). In the current study, the *Proteobacteria*, *Actinobacteriota*, *Bacteroidetes*, *Firmicutes*, *Armatimonadetes*, *Chloroflexi*, and *Acidobacteriia* were the dominant microbial communities in the rice rhizosphere soil (Figure 5), which is consistent with earlier findings (Chen et al., 2021a,b). Our findings revealed that PGPR inoculation could change the rhizobacterial communities (Figure 6), which agree with the previous study (Hu et al., 2020). The rhizobacterial populations were changed after the inoculation of PGPR. The differences in rhizobacterial communities between the inoculated and non-inoculated treatments as well as control, were correlated to growth promotion in a linear method (Figure 7). As a result, the PGPR effect on rhizobacterial populations was typically beneficial to plants, as demonstrated by earlier investigations (Li et al., 2021; Taye et al., 2022). At the genus level, *Streptococcus*, *Paenibacillus*, *Propionispora Rhizobium Couchioplanes*, and *Niastella* showed higher abundance following PGPR inoculation. According to previous research, these taxa were favorably correlated with growth traits and antioxidant activities (Chen et al., 2021b). These bacterial communities are common in plant rhizosphere soil and have the ability to improve plant growth (Kielak et al., 2016; Gu et al., 2020; Chen et al., 2021a). *Bacillus* is the most common member of the *Bacilli* class that has long been employed in microbial fertilizers to boost crop growth and disease resistance (Ali et al., 2021c). Plants have been shown to be protected from abiotic conditions (including cold, salt, and drought) and to have improved nutrition, vigor, and yield when inoculated with *Arthrobacter* and *Bacillus* (Krishnan et al., 2016; Zubair et al., 2019). These findings imply that PGPR inoculation can lead to rhizosphere communities with other beneficial microorganisms (Li et al., 2021).

## Conclusion

The current study discovered that biofilm-forming *Bacillus* strains are resistant to salt stress and have a variety of biological and physiological features that help them cope with salt stress. In comparison to control plants, those treated with bacteria (*Bacillus* strains NMTD17 and GBSW22) demonstrated higher rice plant growth and antioxidant enzyme activity. Biochemical and physiological characteristics were also discovered to play a significant role as a marker of salt stress and can be utilized to determine the efficacy of new bacterial inoculants. Furthermore, inoculation of PGPR strain NMTD17 improved species richness and rhizobacterial abundance, as well as enriched the relative abundances of beneficial bacteria in rice rhizosphere soil. Furthermore, the beneficial effects of *Bacillus* strains in saline environments must be tested in large-scale field trials before they can be used in sustainable agriculture.

## Data availability statement

The datasets generated for this study can be found in NCBI public database. All sequences of 16S *rRNA* genes can be found in Sequence Read Archive (SRA) under BioSample accession no. PRJNA862745.

## Author contributions

QA: conceptualization, investigation, methodology, formal analysis, writing—original draft, and validation. QY, YX and CY: methodology and formal analysis. GM, QG, and HW: validation, investigation, and writing—review and editing. MA, AH, and HM: validation, methodology, and formal analysis. XG: conceptualization, supervision, validation, project administration, and writing—review and editing. All authors contributed to the article and approved the submitted version.

## Funding

This work was supported by the Guidance Foundation of the Sanya Institute of Nanjing Agricultural University (NAUSY-MS18), Fundamental Research Funds for the Central Universities (KYZZ2022001), and the Key Project of NSFC regional innovation and development joint fund (U20A2039).

## Conflict of interest

GM was employed by Shenzhen Batian Ecotypic Engineering Co., Ltd.

The remaining authors declare that the research was conducted in the absence of any commercial or financial relationships that could be construed as a potential conflict of interest.

## Publisher’s note

All claims expressed in this article are solely those of the authors and do not necessarily represent those of their affiliated organizations, or those of the publisher, the editors and the reviewers. Any product that may be evaluated in this article, or claim that may be made by its manufacturer, is not guaranteed or endorsed by the publisher.

## Abbreviations

ACC: 1-Aminocyclopropane-1-carboxylate
APX: Ascorbate peroxidase
CAT: Catalase
DCFH-DA: Sichloro-dihydro-fluorescein diacetate
DEGs: Differentially expressed genes
LB: Luria Bertani
OD: Optical density
PBS: Phosphate buffered solution
PCoA: Principal coordinate analysis
PCR: Polymerase chain reaction
PGP: Plant growth promotion
PGPB: Plant growth-promoting bacteria
PGPR: Plant growth-promoting rhizobacteria
POD: Peroxidase
ROS: Reactive oxygen species
SOD: Superoxide dismutase

## References

[ref1] Abbas R. Rasul S. Aslam K. Baber M. Shahid M. Mubeen F.. (2019). Halotolerant PGPR: a hope for cultivation of saline soils. J. King Saud Univ. 31, 1195–1201. doi: 10.1016/j.jksus.2019.02.019

[ref2] Akram W. Aslam H. Ahmad S. R. Anjum T. Yasin N. A. Khan W. U.. (2019). Bacillus megaterium strain A12 ameliorates salinity stress in tomato plants through multiple mechanisms. J. Plant Interact. 14, 506–518. doi: 10.1080/17429145.2019.1662497

[ref3] Ali M. Ahmad Z. Ashraf M. F. Dong W. (2021a). Maize endophytic microbial-communities revealed by removing PCR and 16S rRNA sequencing and their synthetic applications to suppress maize banded leaf and sheath blight. Microbiol. Res. 242:126639. doi: 10.1016/j.micres.2020.126639, PMID: 33191104

[ref4] Ali Q. Ahmar S. Sohail M. A. Kamran M. Ali M. Saleem M. H.. (2021c). Research advances and applications of biosensing technology for the diagnosis of pathogens in sustainable agriculture. Environ. Sci. Pollut. Res. 28, 9002–9019. doi: 10.1007/s11356-021-12419-6, PMID: 33464530

[ref5] Ali M. Ali Q. Sohail M. A. Ashraf M. F. Saleem M. H. Hussain S.. (2021b). Diversity and taxonomic distribution of Endophytic bacterial Community in the Rice Plant and its Prospective. Int. J. Mol. Sci. 22:10165. doi: 10.3390/ijms221810165, PMID: 34576331 PMC8465699

[ref6] Ali Q. Ayaz M. Yu C. Wang Y. Gu Q. Wu H.. (2022). Cadmium tolerant microbial strains possess different mechanisms for cadmium biosorption and immobilization in rice seedlings. Chemosphere 303:135206. doi: 10.1016/j.chemosphere.2022.135206, PMID: 35660052

[ref7] Allen M. A. Lauro F. M. Williams T. J. Burg D. Siddiqui K. S. De Francisci D.. (2009). The genome sequence of the psychrophilic archaeon, *Methanococcoides burtonii*: the role of genome evolution in cold adaptation. ISME J. 3, 1012–1035. doi: 10.1038/ismej.2009.45, PMID: 19404327

[ref8] Ansari F. A. Ahmad I. (2018). Plant growth promoting attributes and alleviation of salinity stress to wheat by biofilm forming *Brevibacterium* sp. FAB3 isolated from rhizospheric soil. Saudi J. Biol. Sci. doi: 10.1016/j.sjbs.2018.08.003

[ref9] Ansari F. A. Ahmad I. Pichtel J. (2019). Growth stimulation and alleviation of salinity stress to wheat by the biofilm forming Bacillus pumilus strain FAB10. Appl. Soil Ecol. 143, 45–54. doi: 10.1016/j.apsoil.2019.05.023

[ref10] Ayaz M. Ali Q. Farzand A. Khan A. R. Ling H. Gao X. (2021). Nematicidal volatiles from bacillus atrophaeus gbsc56 promote growth and stimulate induced systemic resistance in tomato against meloidogyne incognita. Int. J. Mol. Sci. 22:5049. doi: 10.3390/ijms22095049, PMID: 34068779 PMC8126219

[ref11] Barnawal D. Bharti N. Maji D. Chanotiya C. S. Kalra A. (2014). ACC deaminase-containing Arthrobacter protophormiae induces NaCl stress tolerance through reduced ACC oxidase activity and ethylene production resulting in improved nodulation and mycorrhization in *Pisum sativum*. J. Plant Physiol. 171, 884–894. doi: 10.1016/j.jplph.2014.03.007, PMID: 24913045

[ref12] Bistgani Z. E. Hashemi M. DaCosta M. Craker L. Maggi F. Morshedloo M. R. (2019). Effect of salinity stress on the physiological characteristics, phenolic compounds and antioxidant activity of *Thymus vulgaris* L. and thymus daenensis Celak. Ind. Crop. Prod. 135, 311–320. doi: 10.1016/j.indcrop.2019.04.055

[ref13] Chaves S. Longo M. López A. G. del V Loto F. Mechetti M. Romero C. M. (2020). Control of microbial biofilm formation as an approach for biomaterials synthesis. Colloids Surf. 194:111201. doi: 10.1016/j.colsurfb.2020.111201, PMID: 32615520

[ref14] Chen L. Hao Z. Li K. Sha Y. Wang E. Sui X.. (2021a). Effects of growth-promoting rhizobacteria on maize growth and rhizosphere microbial community under conservation tillage in Northeast China. Microb. Biotechnol. 14, 535–550. doi: 10.1111/1751-7915.13693, PMID: 33166080 PMC7936301

[ref15] Chen L. Li K. Shang J. Wu Y. Chen T. Wanyan Y.. (2021b). Plant growth–promoting bacteria improve maize growth through reshaping the rhizobacterial community in low-nitrogen and low-phosphorus soil. Biol. Fertil. Soils 57, 1075–1088. doi: 10.1007/s00374-021-01598-6

[ref16] Choudhary D. K. (2012). Microbial rescue to plant under habitat-imposed abiotic and biotic stresses. Appl. Microbiol. Biotechnol. 96, 1137–1155. doi: 10.1007/s00253-012-4429-x, PMID: 23073852

[ref17] Compant S. Samad A. Faist H. Sessitsch A. (2019). A review on the plant microbiome: ecology, functions, and emerging trends in microbial application. J. Adv. Res. 19, 29–37. doi: 10.1016/j.jare.2019.03.004, PMID: 31341667 PMC6630030

[ref18] Daliakopoulos I. N. Tsanis I. K. Koutroulis A. Kourgialas N. N. Varouchakis A. E. Karatzas G. P.. (2016). The threat of soil salinity: a European scale review. Sci. Total Environ. 573, 727–739. doi: 10.1016/j.scitotenv.2016.08.177, PMID: 27591523

[ref19] Dogsa I. Choudhary K. S. Marsetic Z. Hudaiberdiev S. Vera R. Pongor S.. (2014). ComQXPA quorum sensing systems may not be unique to Bacillus subtilis: a census in prokaryotic genomes. PLoS One 9:e96122. doi: 10.1371/journal.pone.0096122, PMID: 24788106 PMC4008528

[ref20] Dubey A. Kumar A. Abd-Allah E. F. Hashem A. Khan M. L. (2019). Growing more with less: breeding and developing drought resilient soybean to improve food security. Ecol. Indic. 105, 425–437. doi: 10.1016/j.ecolind.2018.03.003

[ref21] Etesami H. Glick B. R. (2020). Halotolerant plant growth–promoting bacteria: prospects for alleviating salinity stress in plants. Environ. Exp. Bot. 178:104124. doi: 10.1016/j.envexpbot.2020.104124

[ref22] Etesami H. Maheshwari D. K. (2018). Use of plant growth promoting rhizobacteria (PGPRs) with multiple plant growth promoting traits in stress agriculture: action mechanisms and future prospects. Ecotoxicol. Environ. Saf. 156, 225–246. doi: 10.1016/j.ecoenv.2018.03.013, PMID: 29554608

[ref23] Feldman M. Ginsburg I. Al-Quntar A. Steinberg D. (2016). Thiazolidinedione-8 alters symbiotic relationship in *C. albicans*-S. mutans dual species biofilm. Front. Microbiol. 7:140. doi: 10.3389/fmicb.2016.00140 26904013 PMC4748032

[ref24] Gu Y. Dong K. Geisen S. Yang W. Yan Y. Gu D.. (2020). The effect of microbial inoculant origin on the rhizosphere bacterial community composition and plant growth-promotion. Plant Soil 452, 105–117. doi: 10.1007/s11104-020-04545-w

[ref25] Han Q. Ma Q. Chen Y. Tian B. Xu L. Bai Y.. (2020). Variation in rhizosphere microbial communities and its association with the symbiotic efficiency of rhizobia in soybean. ISME J. 14, 1915–1928. doi: 10.1038/s41396-020-0648-9, PMID: 32336748 PMC7367843

[ref26] Hassan A. Amjad S. F. Saleem M. H. Yasmin H. Imran M. Riaz M.. (2021). Foliar application of ascorbic acid enhances salinity stress tolerance in barley (*Hordeum vulgare* L.) through modulation of morpho-physio-biochemical attributes, ions uptake, osmo-protectants and stress response genes expression. Saudi. J. Biol. Sci. 28, 4276–4290. doi: 10.1016/j.sjbs.2021.03.045 PMC8324950 34354410

[ref27] Helmann J. D. Wu M. F. W. Gaballa A. Kobel P. A. Morshedi M. M. Fawcett P.. (2003). The global transcriptional response of Bacillus subtilis to peroxide stress is coordinated by three transcription factors. J. Bacteriol. 185, 243–253. doi: 10.1128/JB.185.1.243-253.2003, PMID: 12486061 PMC141929

[ref28] Hu D. Li S. Li Y. Peng J. Wei X. Ma J.. (2020). Streptomyces sp. strain TOR3209: a rhizosphere bacterium promoting growth of tomato by affecting the rhizosphere microbial community. Sci. Rep. 10, 1–15. doi: 10.1038/s41598-020-76887-5 33208762 PMC7675979

[ref29] Hubbard C. J. Li B. McMinn R. Brock M. T. Maignien L. Ewers B. E.. (2019). The effect of rhizosphere microbes outweighs host plant genetics in reducing insect herbivory. Mol. Ecol. 28, 1801–1811. doi: 10.1111/mec.14989, PMID: 30582660

[ref30] Hussain A. Amna K. M. A. Javed M. T. Hayat K. Farooq M. A. Ali N.. (2019). Individual and combinatorial application of *Kocuria rhizophila* and citric acid on phytoextraction of multi-metal contaminated soils by Glycine max L. Environ. Exp. Bot. 159, 23–33. doi: 10.1016/j.envexpbot.2018.12.006

[ref31] Ke J. Wang B. Yoshikuni Y. (2020). Microbiome engineering: synthetic biology of plant-associated microbiomes in sustainable agriculture. Trends Biotechnol. 39, 244–261. doi: 10.1016/j.tibtech.2020.07.008 32800605

[ref32] Khan M. A. Asaf S. Khan A. L. Adhikari A. Jan R. Ali S.. (2019). Halotolerant rhizobacterial strains mitigate the adverse effects of NaCl stress in soybean seedlings. Biomed. Res. Int. 2019, 1–15. doi: 10.1155/2019/9530963 PMC6925695 31886270

[ref33] Khan W. U. Yasin N. A. Ahmad S. R. Ali A. Ahmed S. Ahmad A. (2017). Role of Ni-tolerant bacillus spp. and *Althea rosea* L. in the phytoremediation of Ni-contaminated soils. Int. J. Phytoremediation 19, 470–477. doi: 10.1080/15226514.2016.1244167, PMID: 27739873

[ref34] Kielak A. M. Cipriano M. A. P. Kuramae E. E. (2016). Acidobacteria strains from subdivision 1 act as plant growth-promoting bacteria. Arch. Microbiol. 198, 987–993. doi: 10.1007/s00203-016-1260-2, PMID: 27339258 PMC5080364

[ref35] Kong M. Sheng T. Liang J. Ali Q. Gu Q. Wu H.. (2021). Melatonin and its homologs induce immune responses via receptors trP47363-trP13076 in *Nicotiana benthamiana*. Front. Plant Sci. 12:691835. doi: 10.3389/fpls.2021.691835, PMID: 34276740 PMC8278317

[ref36] Korenblum E. Dong Y. Szymanski J. Panda S. Jozwiak A. Massalha H.. (2020). Rhizosphere microbiome mediates systemic root metabolite exudation by root-to-root signaling. Proc. Natl. Acad. Sci. 117, 3874–3883. doi: 10.1073/pnas.1912130117, PMID: 32015118 PMC7035606

[ref37] Krishnan R. Menon R. R. Tanaka N. Busse H.-J. Krishnamurthi S. Rameshkumar N. (2016). *Arthrobacter pokkalii* sp nov, a novel plant associated actinobacterium with plant beneficial properties, isolated from saline tolerant pokkali rice, Kerala, India. PLoS One 11:e0150322. doi: 10.1371/journal.pone.0150322, PMID: 26963092 PMC4786123

[ref38] Kumar V. Kumar P. Khan A. (2020b). Optimization of PGPR and silicon fertilization using response surface methodology for enhanced growth, yield and biochemical parameters of French bean (*Phaseolus vulgaris* L.) under saline stress. Biocatal. Agric. Biotechnol. 23:101463. doi: 10.1016/j.bcab.2019.101463

[ref39] Kumar A. Singh S. Gaurav A. K. Srivastava S. (2020a). Plant growth-promoting bacteria: biological tools for the mitigation of salinity stress in plants. Front. Microbiol. 11:1216. doi: 10.3389/fmicb.2020.01216, PMID: 32733391 PMC7358356

[ref40] Kumar A. Singh S. Mukherjee A. Rastogi R. P. Verma J. P. (2021). Salt-tolerant plant growth-promoting Bacillus pumilus strain JPVS11 to enhance plant growth attributes of rice and improve soil health under salinity stress. Microbiol. Res. 242:126616. doi: 10.1016/j.micres.2020.126616, PMID: 33115624

[ref41] Kumar A. Verma J. P. (2018). Does plant—microbe interaction confer stress tolerance in plants: a review? Microbiol. Res. 207, 41–52. doi: 10.1016/j.micres.2017.11.004, PMID: 29458867

[ref42] Kumar A. Verma J. P. (2019). “The role of microbes to improve crop productivity and soil health,” in Ecological Wisdom Inspired Restoration Engineering. eds. Achal V. Mukherjee A. (Berlin: Springer), 249–265.

[ref43] Kumar A. Vyas P. Malla M. A. Dubey A. (2019). Taxonomic and functional annotation of termite degraded (lam.) Kuntze (flame of the Forest). Open Microbiol. J. 13, 154–163. doi: 10.2174/1874285801913010154

[ref44] Li X. Sun P. Zhang Y. Jin C. Guan C. (2020). A novel PGPR strain Kocuria rhizophila Y1 enhances salt stress tolerance in maize by regulating phytohormone levels, nutrient acquisition, redox potential, ion homeostasis, photosynthetic capacity and stress-responsive genes expression. Environ. Exp. Bot. 174:104023. doi: 10.1016/j.envexpbot.2020.104023

[ref45] Li Y. Wang M. Chen S. (2021). Application of N2-fixing Paenibacillus triticisoli BJ-18 changes the compositions and functions of the bacterial, diazotrophic, and fungal microbiomes in the rhizosphere and root/shoot endosphere of wheat under field conditions. Biol. Fertil. Soils 57, 347–362. doi: 10.1007/s00374-020-01528-y

[ref46] Liang Z. Ali Q. Wang Y. Mu G. Kan X. Ren Y.. (2022). Toxicity of *Bacillus thuringiensis* strains derived from the novel crystal protein Cry31Aa with high nematicidal activity against rice parasitic nematode *Aphelenchoides besseyi*. Int. J. Mol. Sci. 23:8189. doi: 10.3390/ijms23158189, PMID: 35897765 PMC9331774

[ref47] Liu H. Brettell L. E. Qiu Z. Singh B. K. (2020). Microbiome-mediated stress resistance in plants. Trends Plant Sci. 25, 733–743. doi: 10.1016/j.tplants.2020.03.014, PMID: 32345569

[ref48] Manghwar H. Hussain A. Ali Q. Liu F. (2022). Brassinosteroids (BRs) role in plant development and coping with different stresses. Int. J. Mol. Sci. 23:1012. doi: 10.3390/ijms23031012, PMID: 35162936 PMC8835148

[ref49] Marsden A. E. Grudzinski K. Ondrey J. M. DeLoney-Marino C. R. Visick K. L. (2017). Impact of salt and nutrient content on biofilm formation by Vibrio fischeri. PLoS One 12:e0169521. doi: 10.1371/journal.pone.0169521, PMID: 28122010 PMC5266276

[ref50] Mengistu A. A. (2020). Endophytes: colonization, behaviour, and their role in defense mechanism. Int. J. Microbiol. 2020, 1–8. doi: 10.1155/2020/6927219 PMC7414354 32802073

[ref51] Mubeen S. Shahzadi I. Akram W. Saeed W. Yasin N. A. Ahmad A.. (2022). Calcium nanoparticles impregnated with benzenedicarboxylic acid: a new approach to alleviate combined stress of ddt and cadmium in brassica alboglabra by modulating bioacummulation, antioxidative machinery and osmoregulators. Front. Plant Sci. 13:825829. doi: 10.3389/fpls.2022.825829, PMID: 35356123 PMC8959818

[ref52] Mukherjee A. Gaurav A. K. Singh S. Chouhan G. K. Kumar A. Das S. (2019). Role of potassium (K) Solubilising microbes (KSM) in growth and induction of resistance against biotic and abiotic stress in plant: a book review. Clim. Chang. Environ. Sustain. 7, 212–214.

[ref53] Mukherjee A. Singh B. K. Verma J. P. (2020). Harnessing chickpea (Cicer arietinum L.) seed endophytes for enhancing plant growth attributes and bio-controlling against *Fusarium* sp. Microbiol. Res. 237:126469. doi: 10.1016/j.micres.2020.126469, PMID: 32251977

[ref54] Narayanasamy S. Thangappan S. Uthandi S. (2020). Plant growth-promoting bacillus sp. cahoots moisture stress alleviation in rice genotypes by triggering antioxidant defense system. Microbiol. Res. 239:126518. doi: 10.1016/j.micres.2020.126518, PMID: 32604045

[ref55] Numan M. Bashir S. Khan Y. Mumtaz R. Shinwari Z. K. Khan A. L.. (2018). Plant growth promoting bacteria as an alternative strategy for salt tolerance in plants: a review. Microbiol. Res. 209, 21–32. doi: 10.1016/j.micres.2018.02.003, PMID: 29580619

[ref56] Ouyang S. Liu Y. Liu P. Lei G. He S. Ma B.. (2010). Receptor-like kinase OsSIK1 improves drought and salt stress tolerance in rice (*Oryza sativa*) plants. Plant J. 62, 316–329. doi: 10.1111/j.1365-313X.2010.04146.x, PMID: 20128882

[ref57] Ramu V. S. Paramanantham A. Ramegowda V. Mohan-Raju B. Udayakumar M. Senthil-Kumar M. (2016). Transcriptome analysis of sunflower genotypes with contrasting oxidative stress tolerance reveals individual-and combined-biotic and abiotic stress tolerance mechanisms. PLoS One 11:e0157522. doi: 10.1371/journal.pone.0157522, PMID: 27314499 PMC4912118

[ref58] Rasul M. Yasmin S. Zubair M. Mahreen N. Yousaf S. Arif M.. (2019). Phosphate solubilizers as antagonists for bacterial leaf blight with improved rice growth in phosphorus deficit soil. Biol. Control 136:103997. doi: 10.1016/j.biocontrol.2019.05.016

[ref59] Sarkar A. Ghosh P. K. Pramanik K. Mitra S. Soren T. Pandey S.. (2018). A halotolerant *Enterobacter* sp. displaying ACC deaminase activity promotes rice seedling growth under salt stress. Res. Microbiol. 169, 20–32. doi: 10.1016/j.resmic.2017.08.005, PMID: 28893659

[ref60] Serra T. S. Figueiredo D. D. Cordeiro A. M. Almeida D. M. Lourenço T. Abreu I. A.. (2013). OsRMC, a negative regulator of salt stress response in rice, is regulated by two AP2/ERF transcription factors. Plant Mol. Biol. 82, 439–455. doi: 10.1007/s11103-013-0073-9, PMID: 23703395

[ref61] Shah A. A. Aslam S. Akbar M. Ahmad A. Khan W. U. Yasin N. A.. (2021). Combined effect of Bacillus fortis IAGS 223 and zinc oxide nanoparticles to alleviate cadmium phytotoxicity in Cucumis melo. Plant Physiol. Biochem. 158, 1–12. doi: 10.1016/j.plaphy.2020.11.011, PMID: 33278679

[ref62] Singh B. K. Trivedi P. Egidi E. Macdonald C. A. Delgado-Baquerizo M. (2020). Crop microbiome and sustainable agriculture. Nat. Rev. Microbiol. 18, 601–602. doi: 10.1038/s41579-020-00446-y 33037425

[ref63] Solangi Z. A. Ali Q. Soomro Z. A. Saleem M. H. Rattar T. M. (2021). Effects of drought stress on morphological, physiological traits of wheat (TriticumAestivumL.) cultivars in Pakistan. J Plant Physiol Pathol 93:2.

[ref64] Song J. Kong Z. Q. Zhang D. D. Chen J. Y. Dai X. F. Li R. (2021). Rhizosphere microbiomes of potato cultivated under *Bacillus subtilis* treatment influence the quality of potato tubers. Int. J. Mol. Sci. 22:12065. doi: 10.3390/ijms222112065, PMID: 34769506 PMC8584837

[ref65] Stassen M. J. J. Hsu S.-H. Pieterse C. M. J. Stringlis I. A. (2020). Coumarin communication along the microbiome–root–shoot axis. Trends Plant Sci. 26, 169–183. doi: 10.1016/j.tplants.2020.09.008 33023832

[ref66] Sun A. Jiao X.-Y. Chen Q. Wu A.-L. Zheng Y. Lin Y.-X.. (2021). Microbial communities in crop phyllosphere and root endosphere are more resistant than soil microbiota to fertilization. Soil Biol. Biochem. 153:108113. doi: 10.1016/j.soilbio.2020.108113

[ref67] Taye Z. M. Noble K. Siciliano S. D. Helgason B. L. Lamb E. G. (2022). Root growth dynamics, dominant Rhizosphere bacteria, and correlation between dominant bacterial genera and root traits through *Brassica napus* development. Plant Soil 473, 441–456. doi: 10.1007/s11104-022-05296-6

[ref68] Trivedi P. Leach J. E. Tringe S. G. Sa T. Singh B. K. (2020). Plant–microbiome interactions: from community assembly to plant health. Nat. Rev. Microbiol. 18, 607–621. doi: 10.1038/s41579-020-0412-1, PMID: 32788714

[ref69] Wang Q. Guan Y. Wu Y. Chen H. Chen F. Chu C. (2008). Overexpression of a rice OsDREB1F gene increases salt, drought, and low temperature tolerance in both Arabidopsis and rice. Plant Mol. Biol. 67, 589–602. doi: 10.1007/s11103-008-9340-6, PMID: 18470484

[ref70] Wang Q. Kang L. Lin C. Song Z. Tao C. Liu W.. (2019). Transcriptomic evaluation of Miscanthus photosynthetic traits to salinity stress. Biomass Bioenergy 125, 123–130. doi: 10.1016/j.biombioe.2019.03.005

[ref71] Wu H. Gu Q. Xie Y. Lou Z. Xue P. Fang L.. (2019). Cold-adapted Bacilli isolated from the Qinghai–Tibetan plateau are able to promote plant growth in extreme environments. Environ. Microbiol. 21, 3505–3526. doi: 10.1111/1462-2920.14722, PMID: 31233661

[ref72] Yao L. Cheng X. Gu Z. Huang W. Li S. Wang L.. (2018). The AWPM-19 family protein OsPM1 mediates abscisic acid influx and drought response in rice. Plant Cell 30, 1258–1276. doi: 10.1105/tpc.17.00770, PMID: 29716991 PMC6048790

[ref73] Yu S. Huang A. Li J. Gao L. Feng Y. Pemberton E.. (2018). OsNAC45 plays complex roles by mediating POD activity and the expression of development-related genes under various abiotic stresses in rice root. Plant Growth Regul. 84, 519–531. doi: 10.1007/s10725-017-0358-0

[ref74] Zhang S. Fan C. Wang Y. Xia Y. Xiao W. Cui X. (2018). Salt-tolerant and plant-growth-promoting bacteria isolated from high-yield paddy soil. Can. J. Microbiol. 64, 968–978. doi: 10.1139/cjm-2017-0571, PMID: 30148967

[ref75] Zhang X. Long Y. Huang J. Xia J. (2020). OsNAC45 is involved in ABA response and salt tolerance in rice. Rice 13, 1–13. doi: 10.1186/s12284-020-00440-1 33284415 PMC7721851

[ref76] Zubair M. Hanif A. Farzand A. Majid T. Sheikh M. (2019). Genetic screening and expression analysis of psychrophilic bacillus spp. Reveal Their Potential to Alleviate Cold Stress and Modulate Phytohormones in Wheat. Microorganisms 7:337. doi: 10.3390/microorganisms7090337, PMID: 31510075 PMC6780275

