# Supplementary material for: Revealing plant growth-promoting mechanisms of Bacillus strains in elevating rice growth and its interaction with salt stress
Source: Front Plant Sci. 2022 Sep 2;13:994902. doi: 10.3389/fpls.2022.994902 (PMC9479341; doi:10.3389/fpls.2022.994902)
Supplement: Supplementary file 1 [file Data_Sheet_1.docx]

***Supplementary file***

Revealing Plant Growth-Promoting Mechanisms of *Bacillus* strains in Elevating Rice Growth and its Interaction with Salt Stress

Qurban Ali^1^, Muhammad Ayaz^1^, Guangyuan Mu^2^, Amjad Hussain^3^, Qiu Yuanyuan^1^, Chenjie Yu^1^, Yujiao Xu^1^, Hakim Manghwar^4^, Qin Gu^1^, Huijun Wu^1^, and Xuewen Gao^1*^

^1^The Sanya Institute of Nanjing Agricultural University, Key Laboratory of Integrated Management of Crop Diseases and Pests, Department of Plant Pathology, College of Plant Protection, Nanjing Agricultural University, Nanjing 210095, China.

^2^Shenzhen Batian Ecotypic Engineering Co., Ltd, Shenzhen, 518057, China.

^3^National Key Laboratory of Crop Genetic Improvement, Huazhong Agricultural University, Wuhan 430070, China.

^4^Lushan Botanical Garden, Chinese Academy of Sciences, Jiujiang 332000, China.

***Corresponding author:** gaoxw@njau.edu.cn (X.G.) Tel: +86-025-8439-5268

**2. Material and Methods**

**2.1 Assessment of ROS in bacterial strain under saline conditions**

The magnitude of cell damage or malfunction is closely linked to the unfavorable conditions of ROS production (Ayaz et al., 2021). ROS was detected in each strain grown under various salt concentrations as an indication of cell damage or cell disturbance. The bacterial strains (NMTD17, GBSW22, and control FZB42) were grown for ROS detection under different salt concentrations on LB medium with different salt concentrations incubated for 96 h at 37°C. After 96 h, the bacterial cells of each strain were harvested by centrifugation at 1000 rpm at 4°C. The harvested cells were then incubated at 25°C for 30 minutes, having a pH of 7.4 with the combination of 10 Mm sodium phosphate buffer and dichloro-dihydro-fluorescein diacetate (DCFH-DA) bought from the company (JianCheng Bioengineering, Nanjing, China) (Massawe et al., 2018). The dye has the ability to stain the ROS-containing material, and the fluorescence was detected using an Olympus 1X71 microscope and the image pro express application v.6.2 (Olympus, Tokyo, Japan).

**2.2 RNA extractions and qPCR**

For total RNA extraction, bacterial strains NMTD17, GBSW22, and control FZB42 were grown for 96 h in a shaker incubator under various salt concentrations (1, 4, 7, 9, 11, 13, and 16 %) in liquid LB culture at 37°C. To observe the expression of the selected salt-resistant genes in each strain, the samples were harvested 96 h after inoculation. The RNA extraction kit (OMEGA Bio-tek, Inc. Norcross, GA, USA) was used to extract RNA from different samples. The extracted RNA purity was determined using Nano-drop (Nano-drop 1000, Thermo Science, Wilmington, DE, USA) by measuring the absorbance at 260/280 nm. The cDNA was synthesized from the isolated bacterial RNA samples of each strain at different salt concentrations in order to perform qPCR and analyze salt-tolerant and non-salt tolerant bacteria. The 5 × All-In-One RT Master Mix (with AccuRT Genomic DNA Removal Kit) by Applied Biological Materials Inc. (abm®, Beijing) was used to synthesize cDNA, as followed by the kit method.

**2.3 Effect on vigor index parameters under salt stress**

The effect of salt stress on the germination of rice and the growth of seedlings was calculated by measuring the vigor index. The seeds were surface sterilized with 3% sodium hypochlorite solution on the surface, followed by 70% ethanol and washed with double-distilled water (ddH_2_O). The sterilized seeds were inoculated with *Bacillus* strains NMTD17 (highly tolerant) and GBSW22 (moderately tolerant) and the well-known salt-sensitive bacterial strain FZB42 to determine the growth of rice plants under salt stress. The seeds grown in without salt treatments were used as control (CK), which were treated with simple double distilled water (ddH_2_O). The inoculated seeds were transferred to sterilized petri dishes (9 cm) containing 0.4% agar with various salt treatments (0, 100, 150, and 200 mmol) and incubated in the dark at 25°C. When radical’s germination was half of the length of the shoot, the germination was observed. Each of the five germinated seedlings were then transferred to petri plates containing 0.3% water agar with different salt concentrations and allowed to grow at 25°C for 7 d to detect the influence of salt treatment on the seedling stage in the presence of bacterial strains. Vigor index and germination factors were examined with the formula proposed by Rasul et al. (2019).

**2.4 Root morphology under salt stress**

The ability of PGPR inoculated rice seedlings to grow under different salt concentrations in petri dishes containing 0.3% agar water at 25°C for 7 d was determined. The seeds were surface sterilized thoroughly and inoculated with different PGPRs (NMTD17, GBSW22, and FZB42). The simple ddH_2_O was used as a control CK. The seeds were then allowed to germinate and transferred to petri dishes containing different salt concentrations and allowed to grow at 25°C for 7 d. The seedlings were then removed from each treatment to measure different root parameters to study the root morphology. The root morphological parameters, such as root diameter, length, volume, tips, and surface area were measured using a rhizoscanner (EPSON Perfection V700 Photo, Epson America, Long Beach, CA, USA) and WinRHIZO software given by Regent Instruments Co. (Sainte-Foy, Quebec, Canada) (Rasul et al., 2019).

**2.4 Root morphological analysis by rhizo-scanning**

In control and PGPR inoculated plants under stress conditions, root morphological analysis is mostly performed to find their roles in stress environments. To examine the effect of PGPRs (NMTD17, GBSW22, and FZB42) on rice root morphology in the presence of salt stress, an automated rhizoscanner system was used to measure different morphological root parameters for each rice plant. After 9 d of inoculation, three rice plants were randomly selected for rhizo-scanning from each treatment three times. The mean values were determined for the parameters mentioned above to observe the effects of each PGPR on the morphology of the rice root under salt stress.

**2.5 DNA extraction, 16Sr DNA gene sequencing, and bioinformatics analysis**

Rhizosphere soil samples were collected from PGPR inoculated and un-inoculated rice plants grown under various salt treatments (0, 100, 150, and 200 mmol). The genomic DNA was isolated using applied protocol TIANamp Soil DNA Kit (TIANGEN Biotech (Beijing) Co., Ltd). The V5–V7 portions of the bacterial 16S rRNA gene were amplified using primers (799F: AACMGGATTAGATACCCKG, and 1193R: ACGTCATCCCCACCTTCC). The PCR was performed by following the procedure, i.e., 3 min at 95°C, 27 cycles of 30 seconds at 95°C, 30 seconds at 55°C, and 45 seconds at 72°C, followed by 10 min at 72°C. Three times in a 20 µL mixture having 4 µL of FastPfu Buffer, 0.4 µL of FastPfu polymerase, 0.8 µL of each primer (5 M), 2 µL of 2.5 mM dNTPs, and 10 ng of template DNA. The product was extracted from a 2% agarose gel according to the manufacturer's protocol, using the AxyPrep DNA Gel Extraction Kit (Axygen Biosciences, Union City, CA, USA) and QuantiFluorTM-ST (Promega, Madison, WI, USA).

**Table S1.** Primers used in this study for qPCR in *Bacillus* strains.

| **Gene Name** | **Primer name** | **Primer sequence (5’-3’)** |
| --- | --- | --- |
| *DegU* | D*egU* -F | TCTATCTATTCATGACGATG |
|  | *DegU* -R | GTCGGAATTCATTTACTAAG |
| *HPII* | *HPII*-F | CGGCGAATTACGAACCGAAC |
|  | *HPII*-R | TGCTCAAATGGCGTCTGACT |
| *OpuD* | *OpuD-*F | TCTGCGCAAACAGGTCTTGA |
|  | *OpuD*-R | AACGGAAGCTCATGCTTGGT |
| *ComA* | *ComA* -F | GAGTTGAAGGACAAGTAGCC |
|  | *ComA* -R | GAATCGCAGAGGTCATCATC |
| *SodA* | *SodA* -F | GGTCAAATGCTTCGTCTTATTG |
|  | *SodA* -R | AGAGCTAGAAGAGGGATGAC |
| *OpuAC* | *OpuAC* -F | ATACATATACAGGCATTCAG |
|  | *OpuAC* -R | CAGAATCAGACTTCCTAATC |
| *SodB* | *SodB-*F | CTTTTGGCAGCTTTGCCGAT |
|  | *SodB-*R | AGGACGTGCATTGCGATAGT |
| *DegS* | *DegS* -F | GAGGAAATCCGCAATGCTTATG |
|  | *DegS -*R | CCTTCAGGTCGAACCCTTTAC |

**Table S2.** Primers used in this study for qPCR in rice plants.

| **Gene name** | **Primer name** | **Forward primers sequence (5’-3’)** |
| --- | --- | --- |
| *OsCYP89G1* | *OsCYP89G1*-F | ATGGCGCTTCTCCTGCTCCTCG |
|  | *OsCYP89G1*-R | GAAGAGGTTGCCAATGAGCGGC |
| *OsDREB1F* | *OsDREB1F*-F | CTGCCGTGTTCGTGGACGAGGAC |
|  | *OsDREB1F*-R | ATACATGTCAGTCCATCCATAGCTT |
| *OsEREBP2* | *OsEREBP2*-F | TCGGAGTCGAGCTATCACCA |
|  | *OsEREBP2*-R | AATCTGCGACGTCCATCTCC |
| *OsERF104* | *OsERF104*-F | CACTTCAAACTCACACCGAGACG |
|  | *OsERF104*-R | TACTCCTGGTTGCCTCCCAT |
| *OsSAMDC2* | *OsSAMDC2*-F | AGGAGATAGATGGCACAGA |
|  | *OsSAMDC2*-R | AAGAACATTGGTCATAGCATATAC |
| *OsLEA3–1* | *OsLEA3–1*-F | AATGATTTCCCTTTGGGTC |
|  | *OsLEA3–1*-R | CATCAGTACACATCACCCA |

**
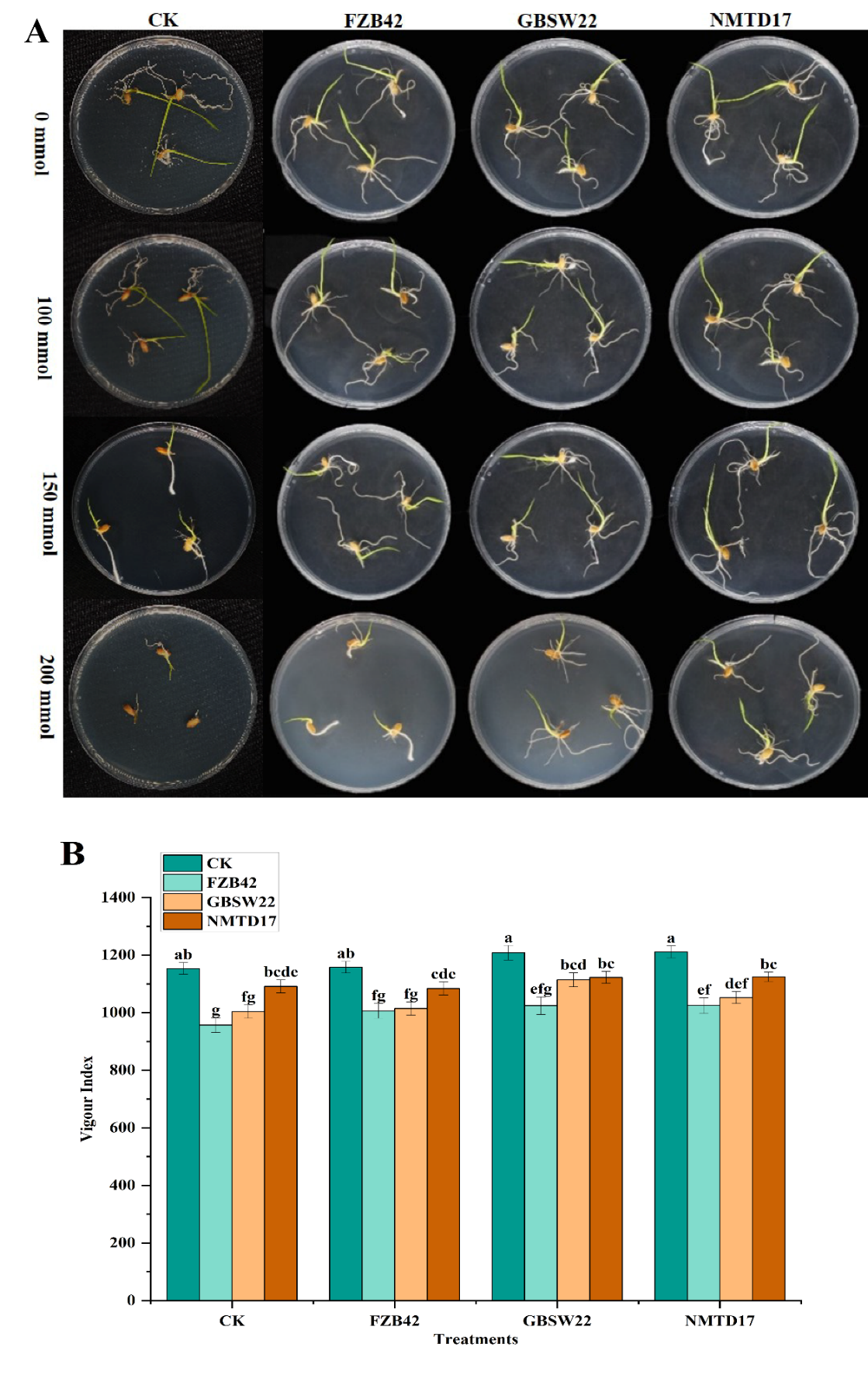
**

**FIGURE S1 |** The rice seeds treated with *Bacillus* strains grown for 7 days under different salt conditions (0, 100, 150, and 200 mmol). (A) Showing the effect of selected strains on rice growth promotion. (B) The graphical illustration of rice seedling VI of inoculated seeds in comparison to control seeds that is used to determine germination percentage and seedling length.

**
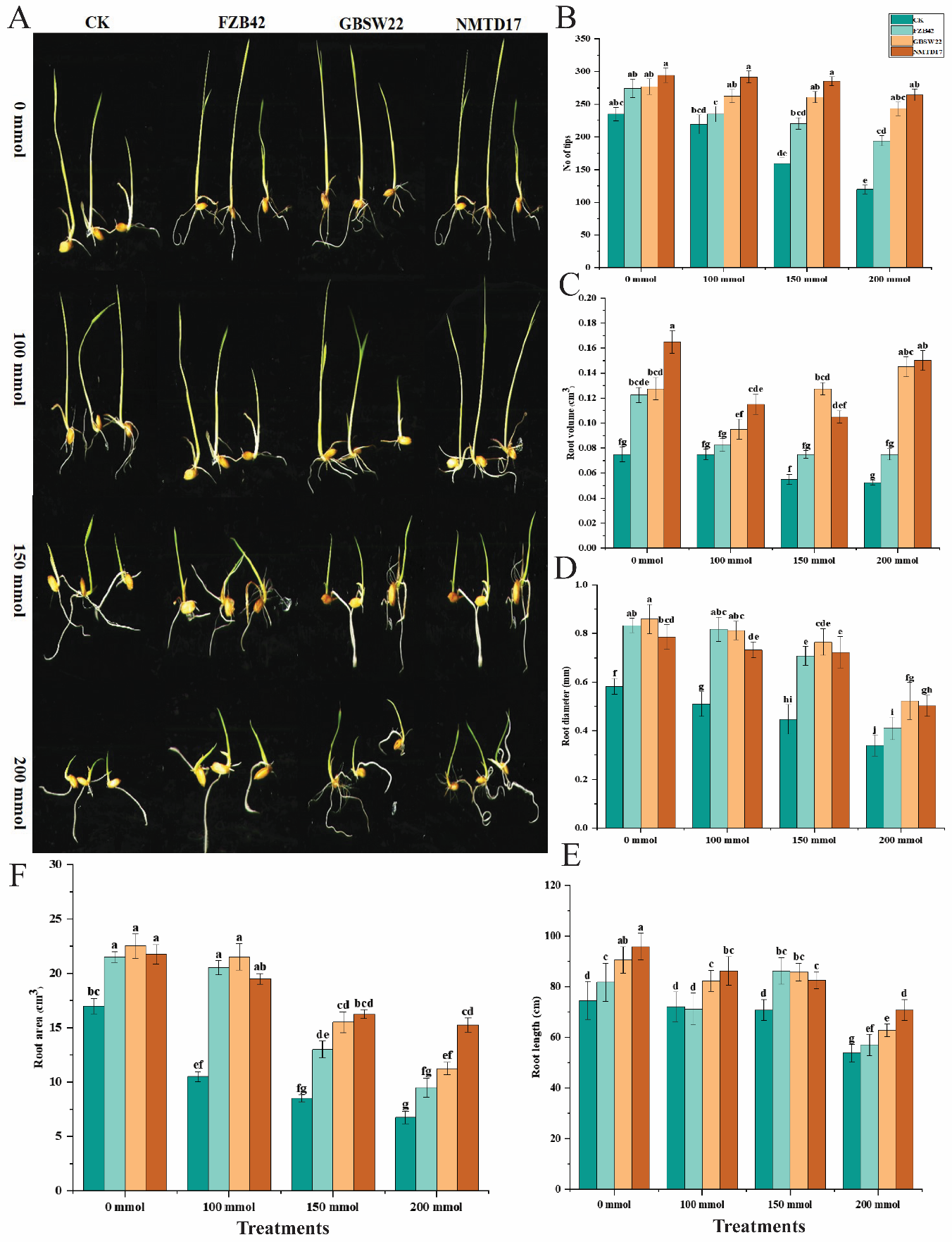
**

**FIGURE S2|** The Rhizo-scanning of seedlings grown under various salt conditions (0, 100, 150, and 200 mmol) inoculated with *Bacillus* strains. (A) Rhizo-scanning of seedlings treated with bacterial strains grown under salt stresses. (B) Graphical representation of root morphological parameters, such as (B) root volume (C) root area (mm^2^), (D) root length (mm), (E) root diameter (cm), and (F) number of root tips. Using Tukey's HSD test, a significant difference between the treatments was found at P ≤ 0.05.

**
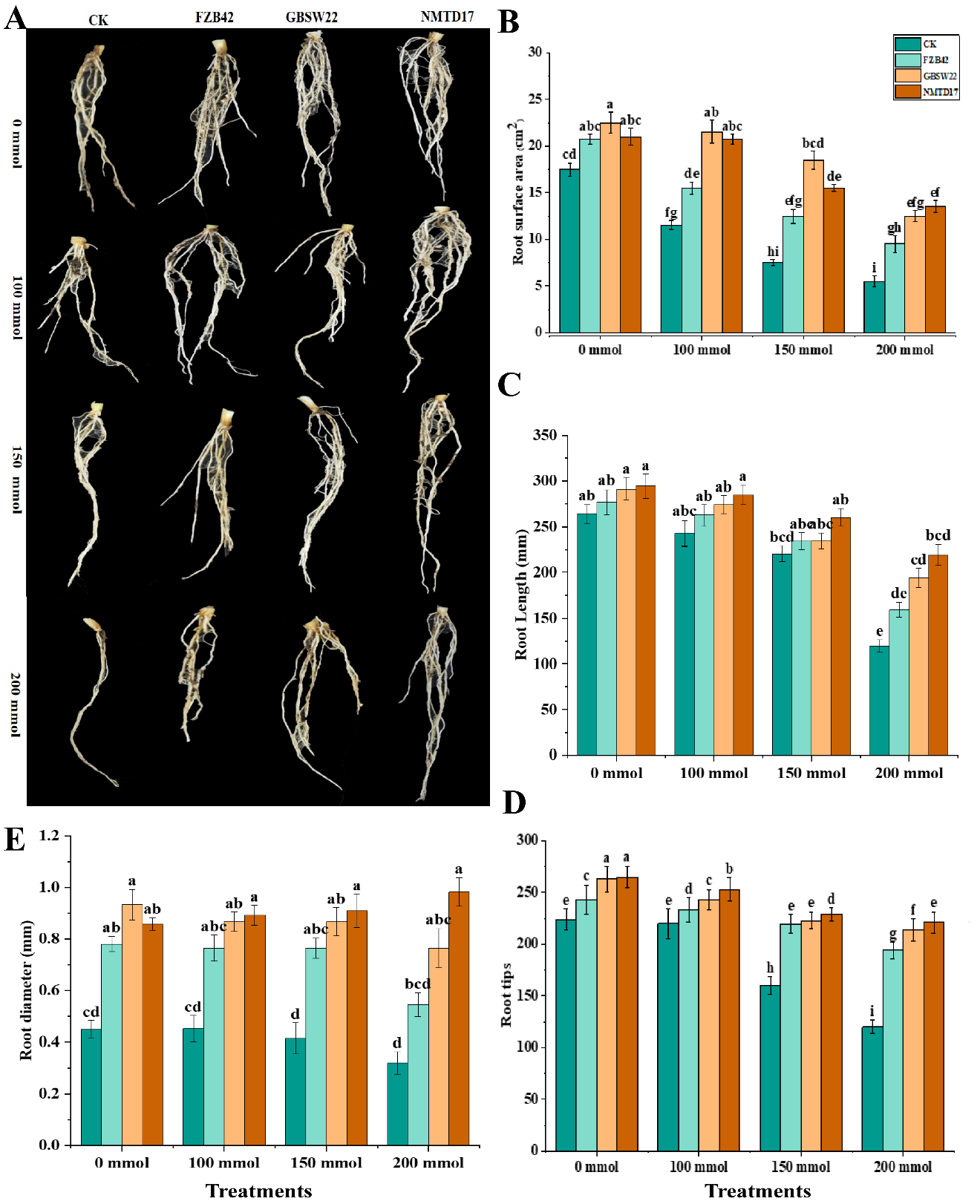
**

**FIGURE S3 |** The Rhizo-scanning of rice plants treated with *Bacillus* sp. grown under various saline conditions (0, 100, 150, and 200 mmol) for 15 days in a pot experiment. (A) The effect of *Bacillus* strains on root morphology, (B) root morphological parameters, such as several root tips for each treatment, (C) diameter of the roots (mm), (D) total root length (mm) of rice plants, (E) total surface area (cm^2^) of roots. Different letters on bars represent the significant differences at P ≤ 0.05 within the treatments at each salt level or among salt levels for each treatment. Vertical bars on graphs indicate the standard deviation of the mean (n=3).


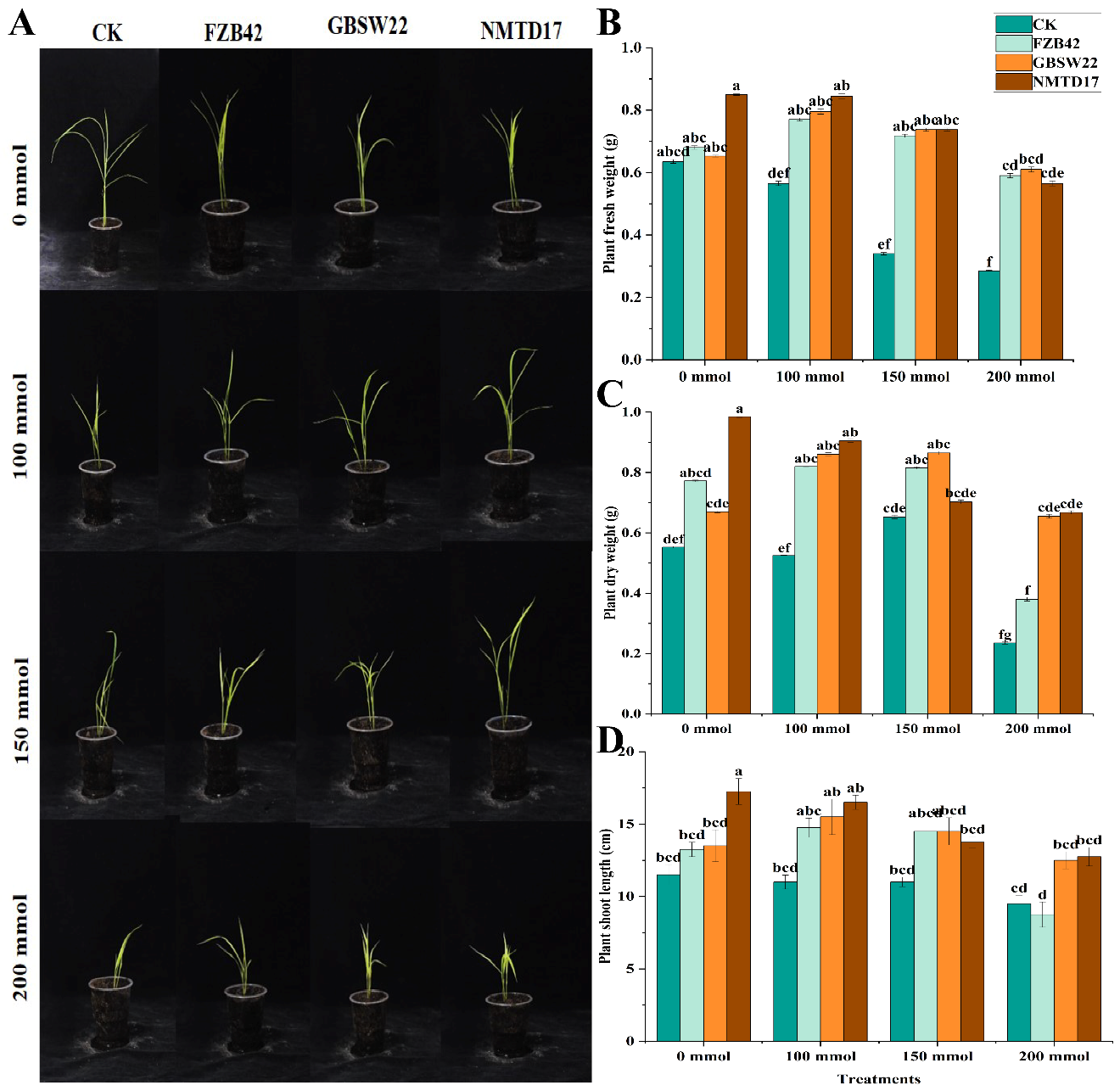


**FIGURE S4 |** The effect of *Bacillus* strains on rice plants grown under different saline conditions (0, 100, 150 and 200 mmol). (A) The effect of specific strains on salt-stressed rice plants. The graphical representation of various growth-promoting factors. (B) shoot length, (C) dry weight, and (D) fresh weight of rice plants with different bacterial treatments. Different letters on bars represent the significant differences at P ≤ 0.05 within the treatments at each salt level or among salt levels for each treatment. Vertical bars on graphs indicate the standard deviation of the mean (n=3).


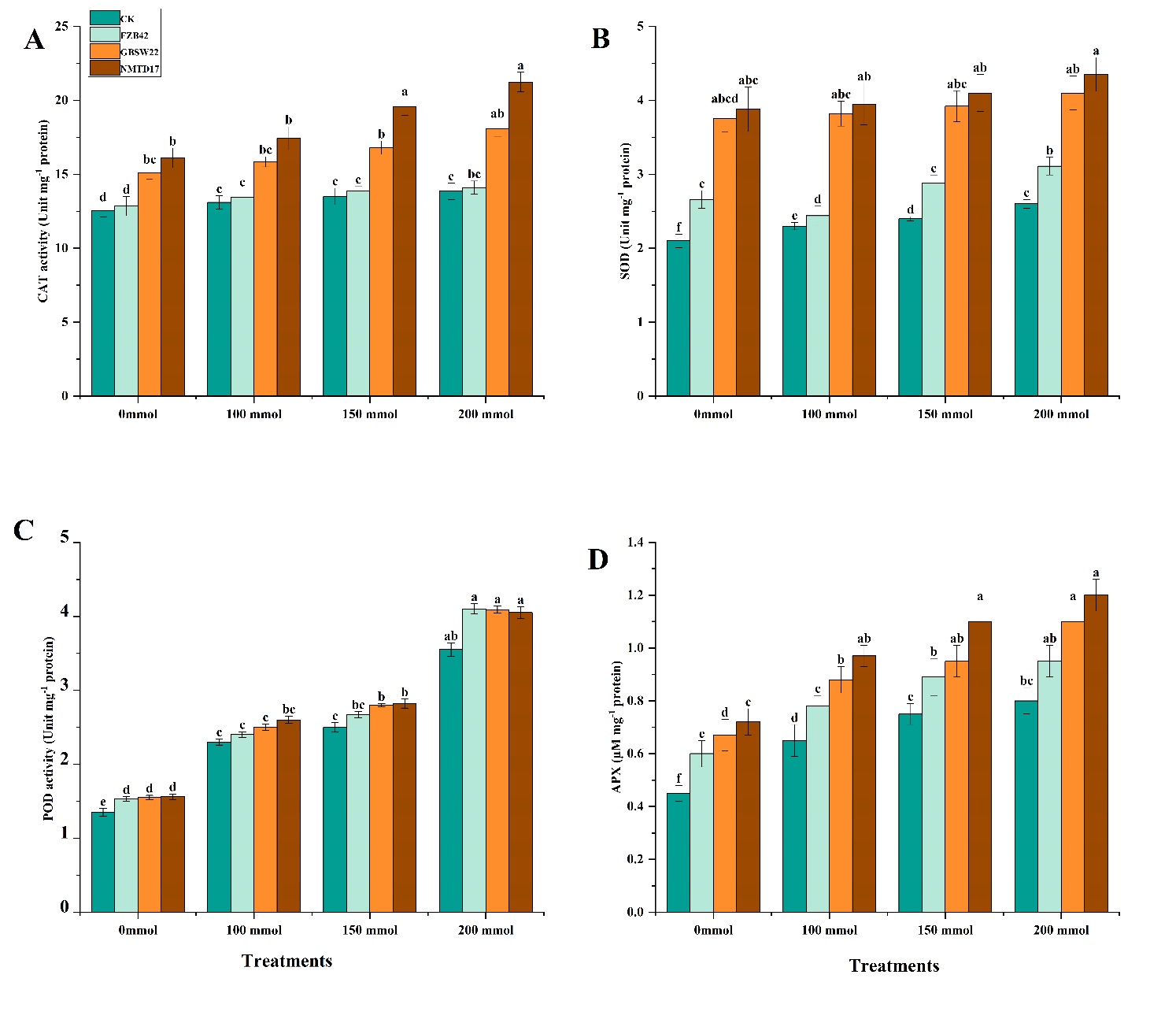


**FIGURE S5 |** Effect of *Bacillus* strains on antioxidative enzymatic activities. (A) Peroxidase (POD, (B) catalase (CAT), (C) superoxide dismutase (SOD), and (D) ascorbate peroxidase (APX). Different letters on bars represent the significant differences at P ≤ 0.05 within the treatments at each salt level or among salt levels for each treatment. Vertical bars on graphs indicate the standard deviation of the mean (n=3).

**Reference**

Ayaz, M., Ali, Q., Farzand, A., Khan, A.R., Ling, H., Gao, X., 2021. Nematicidal volatiles from bacillus atrophaeus gbsc56 promote growth and stimulate induced systemic resistance in tomato against meloidogyne incognita. Int. J. Mol. Sci. 22. https://doi.org/10.3390/ijms22095049

Massawe, V.C., Hanif, A., Farzand, A., Mburu, D.K., Ochola, S.O., Wu, L., Tahir, H.A.S., Gu, Q., Wu, H., Gao, X., 2018. Volatile compounds of endophytic Bacillus spp. have biocontrol activity against Sclerotinia sclerotiorum. Phytopathology 108, 1373–1385.

Rasul, M., Yasmin, S., Zubair, M., Mahreen, N., Yousaf, S., Arif, M., Sajid, Z.I., Mirza, M.S., 2019. Phosphate solubilizers as antagonists for bacterial leaf blight with improved rice growth in phosphorus deficit soil. Biol. Control 136, 103997.
